# Nanomagnetic Guidance Shapes the Structure–Function Relationship of Developing Cortical Networks

**DOI:** 10.1021/acs.nanolett.4c03156

**Published:** 2024-10-21

**Authors:** Connor L. Beck, Conner T. Killeen, Sara C. Johnson, Anja Kunze

**Affiliations:** † Department of Electrical and Computer Engineering, Montana State University, Bozeman, Montana 59717, United States; ‡ Department of Microbiology, Montana State University, Bozeman, Montana 59717, United States; § Optical Technology Center, Montana State University, Bozeman, Montana 59717, United States; ∥ Montana Nanotechnology Center, Montana State University, Bozeman, Montana 59717, United States

**Keywords:** Magnetic nanoparticles, Microelectrode arrays, Electrophysiology, Axon guidance, Neural networks, Neural circuit guidance

## Abstract

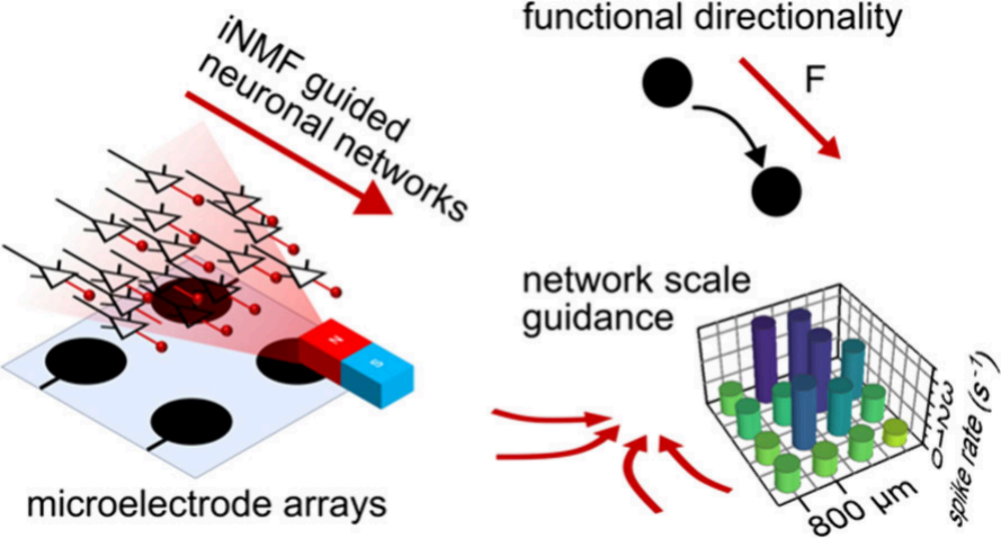

In this study, we implement large-scale nanomagnetic guidance on cortical neurons to guide dissociated neuronal networks during development. Cortical networks cultured over microelectrode arrays were exposed to functionalized magnetic nanoparticles, followed by magnetic field exposure to guide neurites over 14 days *in vitro*. Immunofluorescence of the axonal protein Tau revealed a greater number of neurites that were longer and aligned with the nanomagnetic force relative to nonguided networks. This was further confirmed through brightfield imaging on the microelectrode arrays during development. Spontaneous electrophysiological recordings revealed that the guided networks exhibited increased firing rates and frequency in force-aligned connectivity identified through Granger Causality. Applying this methodology across networks with nonuniform force directions increased local activity in target regions, identified as regions in the direction of the nanomagnetic force. Altogether, these results demonstrate that nanomagnetic forces guide the structure and function of dissociated cortical neuron networks at the millimeter scale.

During neuronal network formation, the brain relies on inputs to construct architectures capable of receiving, processing, and transmitting information.^1−3^ Chemical and mechanical cues polarize neurons through axonal and dendritic specification to establish functional directionality.^4^ Across the neocortex, directionality enables information flow through layered circuitry and long-range connections to produce high-order functions.^5^ As a commonly used analog for cortical circuitry, *in vitro* dissociated cultures provide access to interrogate neuronal function. However, dissociated neuron cultures forfeit the native architecture of the cortex in favor of random wiring, reducing biological relevance and consistency.^6−8^ To mitigate this issue, research has prioritized developing guided *in vitro* cortical circuits.

Engineering dissociated neuronal network topology during development necessitates neurite outgrowth guidance. Various methods achieve neurite guidance through chemical gradients, topographical constraints, or mechanical cues.^9^ Chemical gradients harness biochemical mechanisms that guide axons or promote specification to encourage guided growth.^10^ For example, axonal growth follows gradient cues such as netrin.^11^ Engineering the spatial features of surface adhesion molecules or chemical gradients on substrates can also enable precise formations of networks through microfluidic guidance^12−14^ and microcontact printing.^15,16^ To introduce axonal directionality through surface patterning, polygonal features of poly-l-lysine/laminin with acute patterned angles^17^ or spatial gradients^18^ promote axonal outgrowth and specification. Chemo-repulsive signals like Semamorphin-3F also redirect axonal growth.^19^ Topographically defined circuits are physically guided within structures such as microchannels,^20−23^ microgrooves,^24,25^ or hydrogel^12,13,26−29^ features designed to constrain growth. Directionality is introduced in neuronal circuits cultured inside microfluidic chambers through angled polygonal interconnections between chambers.^30,31^ To further constrain directional connectivity, nanoscale topographical features restrict synaptic directionality.^32^ Lastly, mechanically guided growth relies on physical, force-mediated cues to direct cytoskeletally driven outgrowths. Membrane tension through micropipette pulling drives axonal outgrowth over hours through stretch growth.^33^ Force-mediating technologies require precise spatiotemporal control to draw out neurites over hours with piconewton forces to avoid cytosolic rupture.^34^ Numerous tools have been developed to generate localized forces for mechanical neurite guidance.^35^ For example, Magdesian et al. used an atomic force microscope-guided beads to attach neurites to isolated neurons, resulting in synaptic connectivity.^36^ Similarly, optical or magnetic tweezers can guide neurite outgrowth and connectivity.^37−39^ The precision necessary to generate piconewton forces restricts the expansion of these tools across larger regions, consequently limiting the applicability for network structuring.

Force guidance can be expanded over networks through engineered magnetic fields to provide precise interactions through magnetic nanoparticle transduced forces. This large-scale magnetic gradient manipulation of magnetic nanoparticles, labeled nanomagnetic forces, induces neurite elongation and axonal specification during long-term (≥1 day) forces.^40−43^ Force guidance through magnetic nanoparticles can be mediated through cytosolic^44,45^ or membrane^46^ driven signaling cascades, providing flexibility for the engineering of nanomaterial interactions. To achieve cytosolic force-guidance, magnetic nanoparticles are allowed to enter the cell through endocytosis,^47−49^ then pulled through magnetic gradient forces.^50^ At the millimeter scale, permanent magnets can be arranged to generate low piconewton nanomagnetic forces across neuronal networks.^41^ This flexibility of remote guidance through force-mediating magnetic nanoparticles holds great promise for guiding dissociated neuronal networks. However, it remains to be seen if such guidance can engineer the functionality of networks.

In this study, we introduce large-scale nanomagnetic force guidance to promote aligned functional connectivity in dissociated cortical rat neurons (E18) cultured over microelectrode arrays (MEAs). We observe significant elongation of neurites in the direction of the nanomagnetic force over 14 days *in vitro* (DIV), resulting in network-wide spike rate increases. Furthermore, our analysis using Granger causality indicates a significant alignment in functional connectivity with the magnetic force vector under low-piconewton nanomagnetic forces across the network. This technology can also induce localized regions of high spike rates by utilizing nonuniform force patterns. Altogether, these findings contribute to the knowledge on dissociated network structuring by elucidating the functional responses of force-mediated guidance across networks.

## Nanomagnetic Force Guidance of Neurite Networks

**Figure 1:**
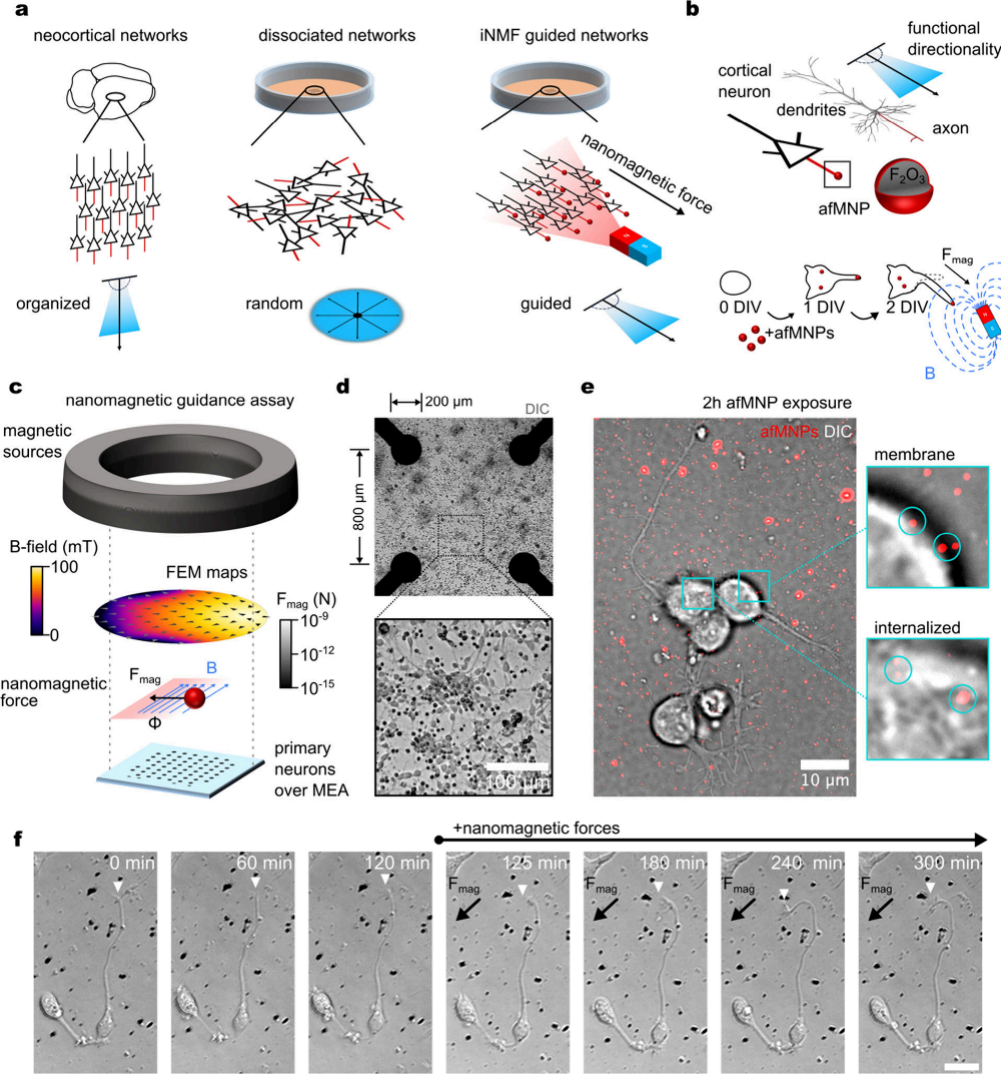
Force-mediating magnetic nanoparticle guidance of primary cortical neuronal networks cultured over microelectrode arrays. (a) Schematic of representative structure–function relationship in the neocortex, dissociated, and force-guided neuronal cultures (iNMF = induced nanomagnetic force). (b) Schematic of functional directionality guidance using magnetic gradient forces (F_mag_) to generate nanomagnetic forces through amine functionalized magnetic nanoparticles (afMNPs). (c) Expanded view of the experimental magnetic platform, with permanent (N52) rare earth magnetic sources designed to induce low piconewton-forces by up-conversion of magnetic fields through the afMNPs on seeded cortical neurons. The magnetic fields and corresponding nanomagnetic forces are simulated through finite element modeling (FEM). (d) Large field of view differential interference contrast imaging shows the dense cortical networks used for network guidance. (e) Merged differential interference contrast and false-color fluorescent image of neurons exposed to afMNPs (red) for 2 h on 1 DIV highlights nanoparticle internalization and membrane interactions. (f) Differential interference contrast timelapse images of neurons (2 DIV, 24 h afMNP exposure) under 120 min of spontaneous growth dynamics and followed by 180 min of nanomagnetic force growth guidance (scalebar: 20 μm).

To engineer the structure and function of developing cortical networks, nanomagnetic forces facilitate directional growth in our cultures (Figure 1a). Dissociated cortical neurons primarily follow nearest neighbor connectivity,^51^ wiring to neurons in the general proximity. Directionality can be introduced through nanomagnetic forces, guiding neurites through magnetic field pulling of magnetic nanoparticles (Figure 1b). By providing time for magnetic nanoparticle interactions before employing large-scale magnetic fields, forces are broadcast across the seeded neurons, providing directionality across the assay. As neurons form connections, neuronal activity can be recorded through microelectrode arrays (MEAs), where millimeter-scaled grids of electrodes enable functional recording when neurons are cultured over the surface. We opted for large microelectrode arrays (60 electrodes, 200 μm diameter, 800 μm pitch) that allowed for measurements of a 36 mm^2^ square region, thus requiring spatially engineered magnetic field interactions to be consistently provided over multiple days (Figure 1c and Figure S1). The magnetic field was engineered following previous Halbach array designs^41,43^ around the culture surface to pull magnetic nanoparticles through gradient force (Figure S2–S3). Across the microelectrode arrays, dissociated cortical rat neurons (E18), were dropwise seeded to promote dense cortical networks over the assay (Figure 1d). Dense networks allow for rich temporal dynamics with high variability,^52^ enabling us to validate force-guidance in functionally dynamic networks.

To establish an experimental force-timeline, we followed a previous protocol^42^ by adding magnetic nanoparticles on 1 DIV and applying forces 24 h after exposure. On 2 DIV, filopodia exuding from neuronal somas form into minor neurites, but axonal and dendritic polarization is typically observed from 2 to 7 DIV, indicating the forces could be used to guide minor neurites through nanoparticles localized in the neurites or induce new neurites from nanoparticles localized in the soma. Nanoparticle internalization is driven by physiochemical properties like radius and surface functionalization, with 100 nm amine functionalized starch bionized nanoferrite magnetic particles (afMNPs) known to uptake as endosomes in primary neurons.^53^ Dynamic light scattering (DLS) analysis showed the afMNP hydrodynamic size resided close to vendor specifications with an observable diameter range of 80–200 nm, where 97% of intensity counts were below 200 nm diameter (Figure S4). As aggregated particles exhibit a minimum diameter of 200 nm, this range suggested afMNPs were not prone to aggregation. Further, the surface charge remained neutral, suggesting particle internalization was not surface charge mediated.^54,55^ To confirm the interfacing of afMNPs with neurons, we cultured primary cortical rat neurons on glass substrates for high-resolution imaging. Cortical neurons exposed on 1 DIV to afMNPs for 2 h showed interactions at both the membrane and cytosol (Figure 1e and Figure S5). As axonal specification is promoted under intracellular operated forces,^42,56^ we encouraged afMNP internalization by extending the exposure time to 24 h before removing excess particles through cell wash. After 24 h exposure, afMNPs were observed throughout the cortical neuron cultures, both internalized and membrane associated (Figure S6). Particles localized in both minor neurites and the soma suggest forces were distributed across the neuronal cytosol. Given this positioning, forces could guide neurites from neurite localized afMNP forces or induce new filipodia through somatic localized afMNP forces. To observe the effects of forces, we recorded afMNP-laden neurons on 2 DIV with timelapse imaging over 2 h of spontaneous growth, followed by 3 h of growth under the magnetic field exposure (Figure 1f). Under forces, the neuronal growth cone followed along the direction of force (Supplemental Videos 1–2). These results indicated that the afMNPs with the magnetic platform can enable guided neurite outgrowth.

**Figure 2:**
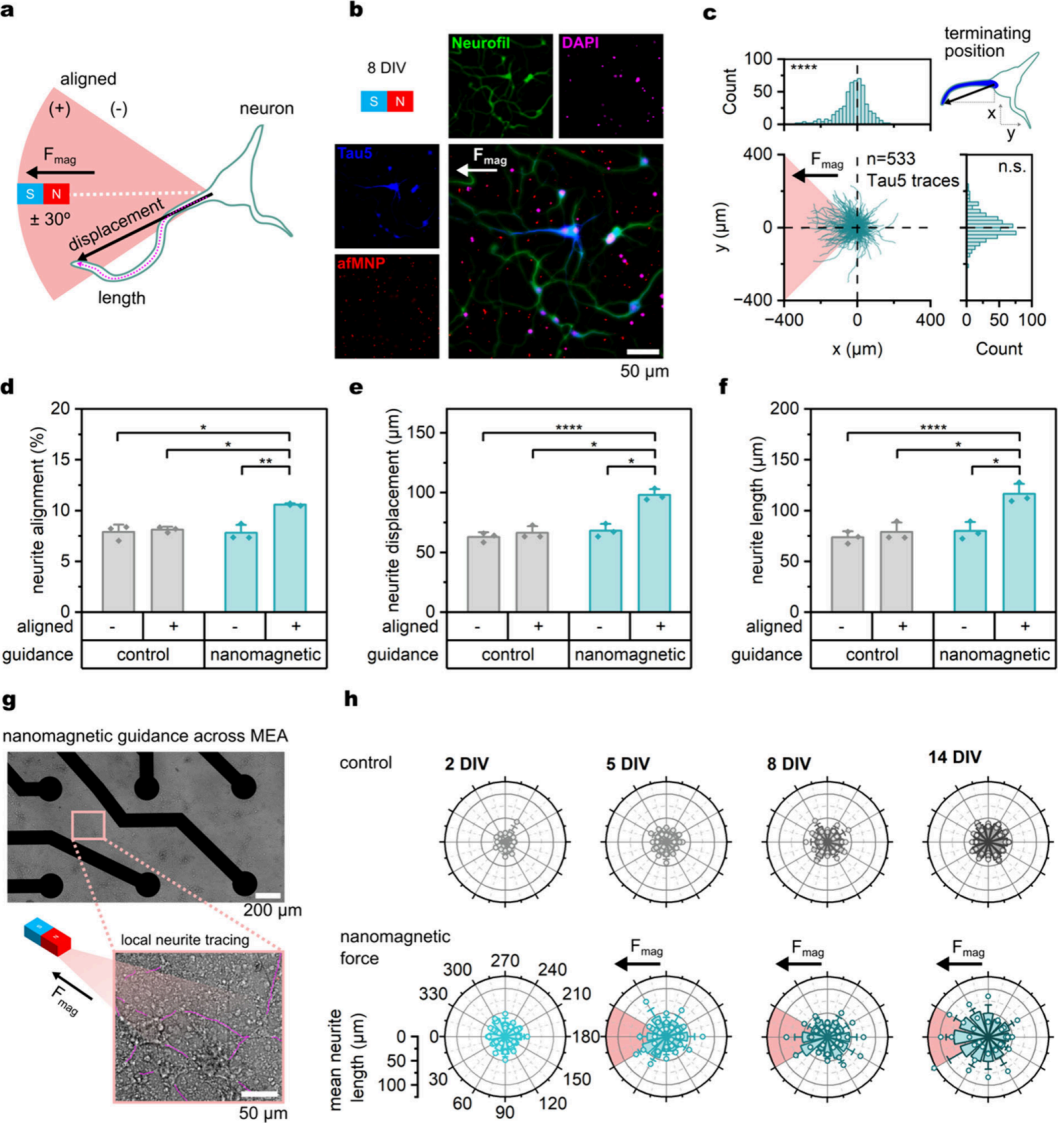
Nanomagnetic forces align neurite directionality. (a) Graphic scheme of neurite guidance parameters. (b) False-colored immunofluorescent image (40x) of nanomagnetic-guided cortical neurons at 8 DIV (Green: Neurofilament (160kD), Magenta: DAPI, Blue: Tau5, Red: afMNPs). (c) Centered traces of Tau5 neurites shows increased frequency of neurites terminating in the direction of the magnetic field (*n* = 1 network). Histograms are counts of the terminating positions of neurites following x and *y* axis. Statistics are 1-sample Wilcoxon’s Signed Rank Test, ****: *p* < 0.0001, ns: *p* > 0.05. (d-f) Neurite characterization of Tau5 specific neurites in control (no afMNPs and no magnetic field) and nanomagnetic force-guided networks (afMNPs and magnetic field). Statistics are 2 sample *t* tests, ****: *p* < 0.0001, ***: *p* < 0.001, **: *p* < 0.01, *: *p* < 0.05, not shown: *p* > 0.05 (*n* = 3 independent networks, > 300 Tau5 neurites per network). (g) Representative brightfield images of primary cortical neurons cultured to 14 DIV across the microelectrode array (black dots and lines) with a representative subregion of neurites traced (magenta lines) relative to the nanomagnetic force direction. (h) Radial mapping of neurite length, where the 0° aligns with the magnetic field vector for control (no afMNPs and no magnet, c) and nanomagnetic force-guided (with afMNPs and magnet, d) networks. Individual circles are the mean neurite length (*n* = 40 traces from 4 subregions in the same network) within a binned angular segment (±30°). Bar plots are the corresponding mean ± SD across 3 separate cultures.

We utilized immunofluorescent staining to further characterize the structural establishment of neuronal networks under nanomagnetic guidance. Cortical cultures contain various cell types that wire together *in vitro* to form dense networks connected functionally through synaptic connections. Mechanical guidance through nanomagnetic forces can mediate neuronal polarity,^42^ indicating connections should follow in the direction of force. Neurite polarization enables the formation of axons through microtubule stabilization with proteins such as Tau. As an essential microtubule associated protein, Tau resides primarily in the axon and is used to assemble and stabilize the microtubule network and act as a mediator of growth cone dynamics. Therefore, force-guidance should present in increased frequency of neurites with Tau. To test this, we cultured cortical neurons under nanomagnetic force guidance, generating 0.4 ± 0.06 pN across the network (Table S1), from 2 to 8 DIV and used immunofluorescence to label Tau5 and medium length Neurofilament (Figure 2a-b). We tracked neurites presenting with Tau5 expression across networks and found a significant increase in neurites terminating in the direction of the magnetic force (Figure 2c). Guided networks presented 60.4 ± 1.8% of neurites terminating in the *x*-direction of the magnetic field, while control networks showed no preference with 49.5 + 3.6% of neurites terminating in the superimposed direction of force. To characterize this further, we binned the number of neurites into 30° bins and identified neurites that terminate within ±30° of the nanomagnetic force direction. The average frequency of Tau5 neurites in the direction of nanomagnetic forces was 10.6 ± 1.3% while nonaligned neurites occurred as 7.8 ± 0.7% within each bin. Control networks (no afMNPs and no magnetic fields) showed no significant directionality preference (Figure 2d). We then analyzed the length and displacement of the Tau5 labeled neurites (Figure 2e-f). Tau5 labeled neurites in the control networks showed no preference with a mean displacement of 64.7 ± 4.7 μm and length of 76.3 ± 0.1 μm. Nanomagnetic-guided networks showed significantly greater displacement in aligned neurites (98.2 ± 4.9 μm) relative to the nonaligned neurites (68.3 ± 5.5 μm) and the control samples, indicating the nanomagnetic forces promoted neurite outgrowth. Tau5 neurite length was similarly greater in the direction of the nanomagnetic force (116.6 ± 9.8 μm) relative to the nonaligned (79.9 ± 8.7 μm) and control neurites.

To validate network structuring on the microelectrode arrays, we exposed afMNP-laden cortical cultures over the microelectrode arrays to the large-scale magnetic fields from 2 to 14 DIV. Neuronal cultures were maintained and imaged on 2, 5, 8, and 14 DIV to track neurite outgrowth with Simple Neurite Tracer.^57^ Given previous results of nanomagnetic force guidance,^42^ we expected longer neurites in the direction of the magnetic field. Therefore, we measured the distance from neurite initiation to tip (Figure 2g). Over the course of 14 DIV, neurons grown independent of a magnetic field showed no directional correlation with a final neurite length of 37.9 ± 16.8 μm (Figure 2h). In contrast, afMNP-guided neurons showed significant alignment over the culture period. On 5 DIV, the length of neurites under nanomagnetic force guidance exhibited increased length compared to control cultures but showed no directionality. The continued forces introduced directionality by 8 DIV, where neurites aligned parallel and antiparallel to the force were significantly longer than those perpendicular to the applied force. We suspect that enhanced neurite length antiparallel to the direction of the force can be attributed to the growth of dendrites in the opposite direction of the axonal differentiation in cells such as pyramidal neurons.^58^ By 14 DIV, neurites aligned parallel to the force exhibited a significantly increased length of 70.5 ± 17.2 μm, while neurites antiparallel exhibited a length of 42.1 ± 4.2 μm (Figure 2d). The reduced neurite length observed here, in comparison to the immunostaining, can be attributed the high density of the networks masking the neurite length. Altogether, force-guided networks exhibited significantly enhanced neurite length across culturing (2-way ANOVA, *p* < 0.0001) with a final length on 14 DIV of 54.5 ± 29.0 μm that was significantly greater than control neurons or afMNP exposed neurons without a magnetic field (Figure S7). To control for image processing bias during neurite selection, we performed fluorescent calcium imaging (Fluo-4AM) of the 14 DIV neurons and applied the Hough transform to identify neurite directionality (Figure S8). A high frequency of crossover regions in the transformed space aligned with the force direction, corresponding to our previous observations, and suggesting neurites were preferentially aligned with the force.^59^ To ensure force guidance did not impact cell viability, we performed a Live/Dead assay and found no significant interactions across experimental parameters (Figure S9). Altogether, these results demonstrate that nanomagnetic forces can structure cortical networks by promoting neurite outgrowth in the direction of the magnetic force.

## Functional Characterization of Nanomagnetic Force-Guided Networks

Cortical networks develop spontaneous spiking by 6 DIV and self-organize into repetitive spiking patterns, progressively maturing with bursting features indicative of network function.^60,61^ We tracked control and nanomagnetic-guided networks during neuronal development from 8 to 14 DIV to investigate the development of network features and neuronal spiking (Figure S10). Neuronal activity was characterized through 4 min passive electrophysiological recording after culturing networks on MEAs (200 μm diameter, 800 μm pitch). Neuronal spiking features were detected from bandpass filtered (300–4000 Hz) signals with a falling edge spike detection (5 standard deviations). The large radius of the electrodes, combined with the high-density cortical networks restrict the use of methods such as electrode spike sorting for single unit analysis. Therefore, detected electrode spikes are indicative of local neuronal population activity. During developmental recordings, we observed significance between DIV in mean firing rate, interspike interval, and mean burst rate (Table S2). Mean firing rate also presented significant interactions between control and nanomagnetic-guided networks, with significantly greater firing rates across the developmental window in nanomagnetic guided-networks. Therefore, we aimed to investigate this further in 14 DIV networks.

**Figure 3:**
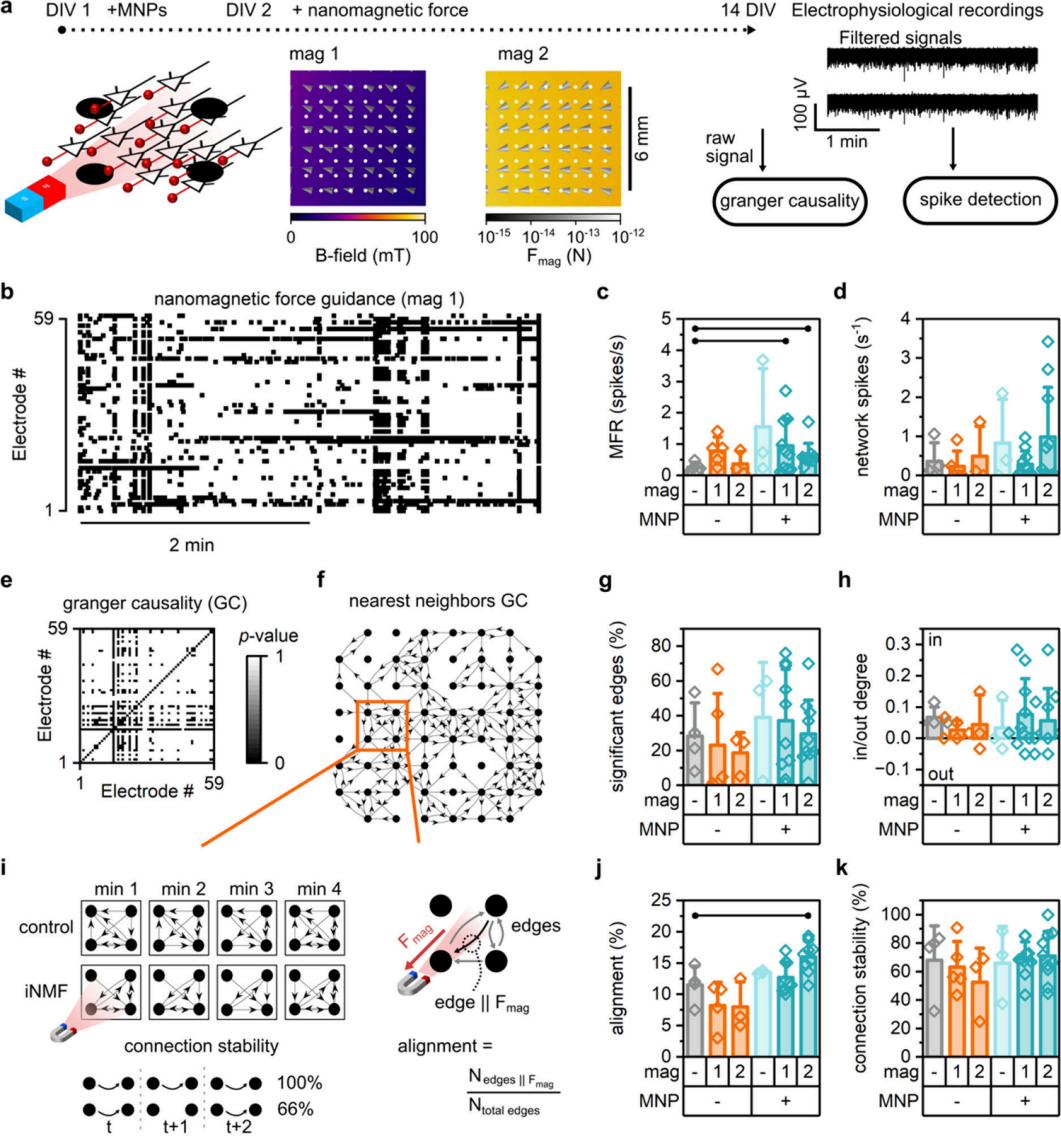
Nanomagnetic force-guided (NMF) circuits exhibit enhanced neuronal activity and aligned directional correlation. (a) Schematic of functional characterization of linear NMF patterned neuronal circuits. Arrowheads on magnetic field maps correspond to the direction of the magnetic gradient force (F_mag_). (b) Example raster plot of a network formed through mag 1 nanomagnetic forces highlights mature features of network synchrony. (c) Mean firing rate (MFR) measured as the mean activity of the 4 min window of recording showed a significant increase in NMF-guided networks in contrast to the controls. (Welch’s test, MNP + mag 1: *p* = 0.0384; MNP + mag 2: *p* = 0.0258). (d) Network spike rate classified as simultaneous spiking on a minimum of 10% of active electrodes within 100 ms windows exhibited no significant changes across networks. (e) Granger causality (GC) used to detect the functional directionality of networks. (f) Graph mapping of GC across the microelectrode array with electrodes plotted as nodes (circles) and edges (lines with arrows) as significant (GC, *p* < 0.05) interactions. Only neighboring (<1200 μm distance) connections were maintained to prioritize the mapping of local information. (g) Schematics of connection stability and alignment metrics to identify key functional parameters. (h) Networks exhibited no significant difference in the number of edges identified by GC. **(i)** Informational flux, measured with in/out degree or the difference of input edges to output edges at a node, showed no significant difference across networks. **(j)** Networks exposed to magnetic fields without MNPs exhibit reduced functional alignment while MNP-exposed cultures exhibited enhanced alignment with the strong magnetic field (Welch’s test, *p* = 0.0478). **(k)** GC detected edges exhibit consistency across networks. All network features were extracted from independent cultured networks (no afMNPs + no mag: *n* = 4, no afMNPs + mag 1: *n* = 5, no afMNPs + mag 2: *n* = 3, afMNPs + no mag: *n* = 3, afMNPs + mag 1: *n* = 9, afMNPs + mag 2: *n* = 9).

To evaluate the functionality of nanomagnetic force-guided networks, we tracked the large-scale network activity patterns through microelectrode array recordings (Figure 3a−b). We implemented two magnetic field patterns, guiding linearly across the MEA with differing magnetic field strengths and corresponding forces (mag 1:22.3 ± 4.1 mT, 0.08 ± 0.02 T/m, 0.20 ± 0.05 pN; mag 2:83.1 ± 1.8 mT, 0.16 ± 0.02 T/m, 0.40 ± 0.06 pN). Control (no afMNP and no magnetic field) networks presented a mean firing rate of 0.26 ± 0.16 spikes/s. Nanomagnetic-guided networks presented significantly greater activity than no magnetic field and no afMNPs across both magnetic field patterns (Figure 3c, MNP + mag 1:0.95 ± 0.87 spikes/s; MNP + mag 2:0.64 ± 0.39 spikes/s). To investigate if network maturation was impacted by nanomagnetic forces, we used synchronous firing (100 ms window with network spike occurring at >10% of electrodes) as a metric of maturity.^62^ We observed no significant deviation in network spiking activity across experimental parameters, indicating that nanomagnetic guidance did not significantly impact the early maturity of the networks (Figure 3d).

An assortment of tools is available to quantify the functional connections of neuronal populations through electrophysiological features. Classically, spike train cross-correlation can identify key correlative features with lag characterization, but it is susceptible to spike-timing changes between neuronal bursting and nonfiring phases.^63^ Substitute methods resolve these susceptibilities by identifying connectivity through correlation metrics such as transfer entropy,^64,65^ mutual information,^66,67^ Granger Causality (GC),^68−70^ or signal coherence.^71^ Here, we implemented pairwise Granger Causal Comparison Analysis (GCCA)^72^ to characterize the directionality of the networks (Figure 3e). In the context of a large-scale, planar microelectrode array, neuronal signals must propagate in linear directions across the array. Hence, GCCA was implemented with nearest neighbors to prioritize these local connections (Figure 3f and Figure S9).

We first examined the connectivity by quantifying the number of significant edges detected across microelectrode arrays by GCCA. Independent of experimental parameters, cortical networks exhibited significant variability in functional connectivity (Figure 3h). To elucidate the stability of network activity, we identified connectivity sources and sinks by measuring the degree difference (in/out degree) of the networks such that electrodes receiving more input correlations than outputs exhibited strong positive in/out degree values. Independent of network structuring, we observed a slightly positive in/out degree (Figure 3i). This indicates that cortical networks are more prone to information sinks; however, network guidance did not impact this functionality.

Given that nanomagnetic pulling should prioritize axonal differentiation in cortical neurons,^73^ we hypothesized that networks would be structured following the nanomagnetic force direction, leading causal connections to be driven more frequently and in the same direction. To address this, we identified the distribution of nanomagnetic aligned connections relative to the total number of connections as a metric of alignment and the reoccurrence of connections as a metric of stability (Figure 3i). Under nanomagnetic force-guidance with mag 2, we observed a greater functional alignment of 16.0 ± 2.5% relative to the 11.5 ± 3.0% alignment exhibited by control networks (Figure 3j). The weaker magnetic potential of mag 1 did not present significant functional alignment relative to the control networks (Welch’s test, *p* = 0.5186), indicating that the lower (0.20 ± 0.05 pN) force pattern may not provide sufficient force to mediate neuronal function. We found no significant difference in the consistency of detected edges across networks, with GC correlations recurring ∼70% of the 4 min recording window (Figure 3k). Taken together, these results indicate that force-guided cortical networks exhibit consistent functional alignment without compromising network functionality.

## Spatially Localized Nanomagnetic Force-Guided Network Activity

**Figure 4:**
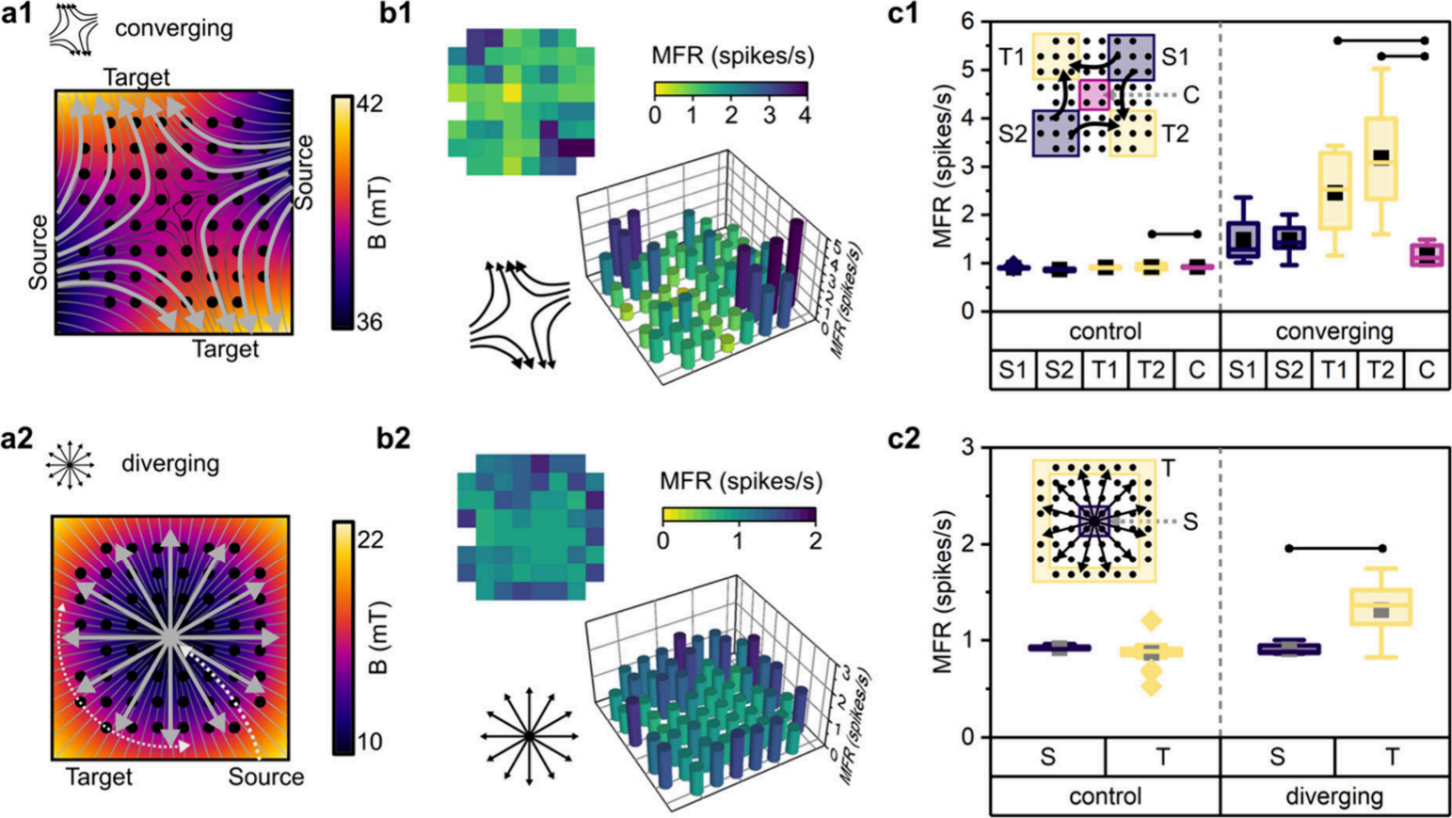
Spontaneous cortical network activity follows gradient-designed nanomagnetic forces. (a1−a2) Finite element modeling of normalized magnetic flux density and gradient lines show pulling directions to engineer (1) converging and (2) diverging networks. (b1−b2) Spatial mapping of the mean firing rate (MFR) across the electrode array highlights strong activity where the network converges or diverges. MFR is computed as the mean spike rate at an electrode over 4 min of spontaneous recording (*n* = 4 independent cultures). (c1−c2) Spatial network activity was categorized by the predicted magnetic field maps into Source (S) and Target (T) regions. Low magnetic gradient regimes were not expected to guide the network and were labeled as constant (C) regions. (c1) Target regions in the converging network exhibited greater activity than the constant region (*n* = 9 electrodes per region; T1: *p* = 0.0040; T2: *p* = 0.0014). (c2) Target regions in diverging network presented increased activity relative to the center source region (*n* = 4 source electrodes, 23 target electrodes; *p* < 0.0001). Solid lines are significant (*p* < 0.05) detections from Welch’s Test.

The flexibility of a fully external magnetic gradient field permits on-hand restructuring of neuronal activity. To examine this, we implemented nonuniform magnetic field gradients to drive spatial shifts in functional activity. We hypothesized that introducing network heterogeneity locally tunes the functional activity patterns within the dissociated cortical networks. To test this, we engineered magnetic field patterns to guide the network with directionally engineered regions labled as source and target regions (Figure 4a1−a2). Target regions are defined as the end points of nanomagnetic guidance within the network, visualized as the higher magnetic potential. Under magnetic field exposure, the afMNPs move along the nanomagnetic force path toward the target regions, generating spatially localized forces to establish the network. The source regions are then defined as the precursor locations to the target regions, such that the afMNPs originating in a source region are pulled to the target region. We aimed to identify the network response by implementing two network designs: (1) a convergent design that guided networks into localized regimes and (2) a divergent design to pull the network out radially. The convergent design also included a central region with minimal gradient force, which we expected would not induce guidance; thus, we labeled it a constant region.

Following our guidance method, cortical networks were exposed to afMNPs and forces to 14 DIV where 4 min spontaneous recordings of the networks were performed. Control networks exposed to afMNPs without guidance presented with 0.90 ± 0.07 spikes/s while both convergent and divergent networks exhibited increased activity with 1.71 ± 0.95 and 1.13 ± 0.28 spikes/s, respectively. By mapping the electrode activity rates, we observed strong activity in the convergent target regions (Figure 4b1). The divergent network furthered this observation, where networks exhibited higher activity around the perimeter of the network than the center (Figure 4b2). To quantify these observations, we binned electrodes into target and source regions and contrasted the spiking activity. The convergent target regions T1 and T2 presented 2.46 ± 0.88 and 3.18 ± 1.16 spikes respectively, nearly double the activity of source regions (1.49 ± 0.53 spikes/s) or the constant, nongradient region of the convergent network (1.17 ± 0.25 spikes/s) (Figure 4c1). The target region of the divergent network exhibited increased activity of 1.31 ± 0.27 spikes/s in the target region relative to the 0.91 ± 0.06 spikes/s detected in the source region (Figure 4c2). Control networks showed minimal variability in the spatial activity profiles, suggesting the local activations were nanomagnetic-guided. These results indicate that nanomagnetic guidance develops local regions of increased activity, which can be enhanced through convergent regions.

Our results demonstrate that nanomagnetic guidance can shape the structure and function of developing networks *in vitro*. The method is simple to employ, requiring a permanent magnetic field applicator and commercially available functionalized magnetic nanoparticles during development. Nanomagnetic guidance expands the growing toolbox of neuronal patterning technologies such as microgrooves,^74−78^ surface patterning,^16,79,80^ or microchannels^20−23,30,81^ by enabling guidance independent of culture surfaces. Contact guidance used with microgrooves requires the fabrication of topographically defined surfaces, limiting the use of recording technologies such as microelectrode arrays. Physical and chemical constraints engineered through surface patterning and microchannels have been employed on microelectrode arrays^16,23,30^ and permit engineering of networks. However, implementing patterning and microchannel methods requires contact aligning and often presents challenges with adhesion. Nanomagnetic guidance circumvents these challenges by enabling remote access to cultures through the magnetic nanoparticles. While we observed a significant increase in guided neurites and functional connectivity, we note the limitations of this technology. An increased frequency of ∼10% observed for Tau labeled neurites and ∼4% for functional alignment indicates the parameters used within experiments here shaped the structure and function of the network, rather than molded it through constraints. Finally, nanomagnetic guidance is not limited to 2-dimensional assays, enabling the spatial engineering of networks in 3-dimensions, which can prove challenging for other technologies.

The directionality of nanomagnetic-guided circuits engineered neuronal activity across a millimeter-scaled dissociated neurite network, indicating this work has great potential to be expanded for the delivery of mechanical cues into tissues, brain organoids,^82^ or stem cell cultures to guide the structure–function relationship. This method’s remote access can be integrated with other technologies such as microfluidic culturing platforms to engineer neuronal circuits through parallelized cues, enhancing the formation of biologically relevant tissue. Given nanomagnetic forces ability to shape the structure and function of networks, future work should prioritize the cellular and circuit-level mechanisms that mediate spiking activity during development. Characterizing synaptic density in guided target regions and long-term synaptic maturation^83,84^ could provide further knowledge into the mechanisms driving neuronal mechanotransduction during development. In summary, this study demonstrates that nanomagnetic force guidance of neurite outgrowth promotes structurally and functionally aligned neuronal networks.

## Supporting Information Available

Supplemental video 1: Time-lapse video of neuronal growth cone following the nanomagnetic force direction over three hours of applied forces (AVI)
Supplemental video 2: Nanomagnetic forces guide growth cones and increase somatic dynamic (AVI)
Materials and methods section and figures showing the magnetic source device, magnetic field simulation and characterization, magnetic nanoparticle characterization, supporting images for neuron-nanomaterial interactions, neurite characterization, Hough transform image processing for supporting neurite directionality, cytotoxicity assay, developmental activity profiling, and nearest-neighbor granger causality metric (PDF)

## Supplementary Material

nl4c03156_si_001.avi

nl4c03156_si_002.avi
