# Supplementary material for: Nanomagnetic Guidance Shapes the Structure–Function Relationship of Developing Cortical Networks
Source: Nano Lett. 2024 Oct 21;24(43):13564–73. doi: 10.1021/acs.nanolett.4c03156 (PMC11529602; doi:10.1021/acs.nanolett.4c03156)
Supplement: Supplementary file 3 — nl4c03156_si_003.pdf [file nl4c03156_si_003.pdf]

Supporting information for: Nanomagnetic guidance shapes the structure-function relationship of developing cortical networks

Connor L. Beck[1], Conner T. Killeen[2], Sara C. Johnson[1], and Anja Kunze[1,3,4,*]

[1]Department of Electrical and Computer Engineering, Montana State University, Bozeman, Montana, 59717, USA

[2]Department of Microbiology, Montana State University, Bozeman, Montana, 59717, USA

[3]Optical Technology Center, Montana State University, Bozeman, Montana, 59717, USA

[4]Montana Nanotechnology Center, Montana State University, Bozeman, Montana, 59717, USA

*Email: anja.kunze@montana.edu

Keywords:
Magnetic nanoparticles, Microelectrode arrays, Electrophysiology, Axon guidance, Neural networks, Neural circuit guidance

This document contains the materials and methods as well as further details about the main article named above. Additional images and graphs are presented to support the results described in the main article. This document contains eleven figures: S1 – S11, two tables: S1 and S2, and two supplemental video descriptions.

Materials and Methods

Magnetic nanoparticle guided cortical networks

To study network guidance, we seeded dissociated primary cortical neurons from E18 rat primary cortical brain tissues over poly-d-lysine (PDL, Gibco) coated microelectrode arrays (MEAs, Multi Channel Systems). Prior to cell culturing, clean microelectrode arrays were coated with PDL overnight followed by a 3x rinse with phosphate buffered saline (PBS, Gibco) and 2 days of incubation (37 ºC, 5 % CO_2) with pretreatment media (90% Neurobasal Plus, 10% Horse Serum, v/v, Gibco). Immediately prior to cell plating, MEAs were aspirated, and neurons (10^6 per dish) were dropwise seeded in the center of the MEA well, above the electrodes. Seeded MEAs were incubated at room temperature for 5 minutes and gently covered with 2 ml of growth media (97% Neurobasal Plus, 2% B27 Plus, 1% Glutamax, v/v, Gibco). Cultured neurons were maintained in incubation at 37 ºC and 5% CO_2. After 24 h, 100 nm diameter, amine terminated starch functionalized magnetic nanoparticles (afMNPs; Micromod, Synomag® BNF-Starch-redF, NH2 surface) were prepared in culture media (96% Neurobasal Plus, 2% B27 Plus, 1% Glutamax, 1% Penicillin-Streptomycin, v/v, Gibco) by sonicating (44 kHz, 37 ºC, 20 min) and added to the cells at 10^{12} particles/ml (5×10^5 particles/cell or 2 µg solid content/mL). The afMNPs were incubated with the neurons for 24 h and then gently washed 2x with pre-warmed culture media (37 ºC) to remove excess nanoparticles. The microelectrode arrays were then placed in the 3D printed magnetic apparatus baseplate and the magnetic ring added over the top and locked in place to ensure a consistent magnetic field exposure over the culture (Figure S1). Cultures were maintained up to DIV 14 with the magnetic field exposed and brightfield imaging was performed at 2, 5, 8, and 14 DIV with culture media exchange every 3-4 days. Following neuronal cell culture and experimentation, the MEAs were cleaned with solvent (Tween 20) and autoclaved after every third use for reuse.

Primary Neuronal Cell Culture

Neuronal cell cultures were established by following previously reported protocols plated on prepared planar-type microelectrode arrays (60ecoMEA, Multi Channel Systems, Germany). In brief, cortical hemispheres (E18, TransnetYX) were dissected from whole tissues in PBS with (33 mM glucose, 1% (v/v) penicillin-streptomycin) and dissociated using 10% (v/v) papain (Carica papaya, Roche, pH 7.3, 15 min, 37 °C) in Hibernate™-E (Gibco). The enzyme solution was then removed and 10% Horse-serum (Gibco) in Neurobasal Plus (Gibco) was added to quench further enzymatic activity. Tissues were then mechanically dissociated through trituration of a 1000 µL pipette tip and filtered through a 40 µL strainer. The dissociated cells were centrifuged (6 min, 500 rpm, at room temperature), resuspended in culture media and seeded at a cell concentration of 1×10^6 cells/mL.

Immunofluorescent labeling

To identify neurites with Tau, cells were cultured at a density of 500,000 cells/mL on 35 mm glass bottom petri dishes, pre-coated with 0.05 mg/mL poly-D-lysine. Control (no afMNP and no magnetic field) and nanomagnetic force guided (afMNPs with magnetic field) samples were cultured to 8 DIV and fixed for antibody staining. Neurons were washed with DPBS (Gibco) and fixed with 4% (v/v) paraformaldehyde in PBS (Bioworld) for 20 min. Cells were washed (DPBS, 3X) then permeabilized for 10 min with 0.1% TritonX (Sigma) and 3% BSA (Sigma) in PBS, then blocked with 3% goat serum (Thermo) and 1% BSA for 30 minutes. The samples were incubated overnight (4°C) with primary antibodies (1:100; mouse anti-Tau5: Invitrogen, MA5-12808; Anti 160 kD Neurofilament Medium antibody, Abcam, NF-09) in 3% goat serum, 0.5% Tween-20 (Sigma) and 1% BSA in DPBS. Following primary antibody application, cells were washed and blocked again with secondary antibodies (1:1000, goat anti-mouse IgG H&L Alexa Fluor 405 Abcam, ab175660; goat anti-rabbit IgG H&L Alexa Fluor 488, Abcam, ab150077) for two hours, followed

by incubation of 1.0 µg/mL DAPI (Sytox Deep Red Nucleic acid stain, Invitrogen, S11381) for 20 minutes and washed with DPBS.

Imaging and neurite tracing

Imaging was performed to characterize afMNP-cell interactions and neurite guidance. All imaging was performed with an inverted microscope (Leica, DMI-8s) with differential interference contrast (DIC) for white light imaging and red fluorescence (TXR filter, 540-580 ex., 592-688 em.) for afMNP imaging. Nanoparticle uptake was tracked optically under 1500x total magnification with oil immersion (NA 1.52). Z-stack images were acquired in both DIC and red fluorescence at 0.2 µm height steps to identify the vertical position of nanoparticles within cells. Images were contrast enhanced in ImageJ with no other alterations. Immunolabeled samples were imaged at 400x total magnification with fluorescent channels: blue (Tau5, 325-375 ex., 435-485 em.), green (160 kD medium Neurofilament, 450-490 ex., 500-550 em.), red (afMNPs, 540-580 ex., 592-688 em.), y5 (DAPI, 590-650 ex., 662-738 em.). 16-bit gray-scale images were analyzed using the Simple Neurite Tracer plugin for FIJI[1]. Blue channel (Tau5) was used to trace neurites. A total of 30 images were acquired for each network with at least 3 cells per image. Tau5 specific neurite traces were exported to MATLAB and traces were mapped for alignment (percentage of neurites within 30º of the nanomagnetic force direction), displacement (Euclidean distance from initiation to termination point), and length (total path length of neurite). To evaluate neurite outgrowth on the MEAs, live-cell differential interference contrast (DIC) imaging was performed with an inverted microscope (Leica, DMI-8 S, 100x total magnification) on 2, 5, 8, and 14 DIV. Five regions across the surface of the MEA were selected to investigate growth directionality. 16-bit gray-scale images were analyzed using the Simple Neurite Tracer plugin for FIJI[1]. A total of 40 traces was acquired within each image. The point where a neurite initiated at the soma to its final position within the field of view were used to compute the directional vector and resulting length.

Acquisition and processing of electrophysiological signals

Electrode signals were acquired with a heat-controlled microelectrode recording platform (MEA2100-Lite, Multi Channel Systems) with data acquisition interface board connected to a computer running Multi Channel Experimenter Software. To minimize neuronal culture responses to environmental shifts, the heat-controlled amplifier was set to 37 ºC and microelectrode arrays were set in the headstage for 5 minutes before recording. Cortical networks were recorded under spontaneous activity for 4 min through the Multichannel experimenter and saved for data processing. A custom MATLAB pipeline was developed for signal processing. In the case of standard signal processing (e.g. Mean firing rate and network spiking), signals were bandpass filtered (300-4000 Hz) and a 5 standard deviation falling edge spike detection was used to extract spikes. A network spike was identified as the co-activity of >10% of the total number of active electrodes in a culture. Co-activity was defined by simultaneous spiking in 100 ms windows. Bursts were detected as a sequence of 10 or more spikes with less than 100 ms between each spike.

Granger causality-based electrophysiological signal processing

To establish functional connectivity, we use a pair-wise Granger causal comparison to correlate raw signals through the open-source toolbox GCCA developed by Seth[2]. The causality was detected through the iterative approach designed within the package and is visualized in Figure S7.

Because of the large electrode radii, we use a nearest neighbors' measure to establish functional connectivity relationships. Only the surrounding electrodes were correlated at a given time. Electrodes were parsed into 9-electrode regions across the MEA based on the center electrode. Edge electrodes were processed with this method with fewer electrodes. Local field potential recordings were collected from each electrode in the region for a single granger measurement. To ensure valid correlations, we first used spike

detection to ensure an electrode was active, otherwise the electrode was excluded from the GCCA computation. After electrodes had been selected, they were cropped into 1-min windows for each correlation measurement.

GCCA was implemented with 10 trials and 1000 observations per trial with an ordinary least squares estimation of the vector autoregression (VAR) model approach and a locally weighted regression (LWR) estimation for the information criteria regression with 5-node test networks. The aikake information criteria (AIC) model order was used with a 20-order maximum. Time-domain conditional Granger causality measurements were calculated for each electrode set. Final significance of a connection during the minute was determined as a mean pairwise-conditional causality less than 0.05 alpha level. Such significant connections were then represented as graphical edges with directionality retained.

Each local nearest neighbor correlation was then restitched together across the network to retain the spatial elements. Significant edges were measured as the number of edges relative to the total maximum. In/out degree was measured as the ratio of input to output edges at a given node. For alignment measurements, the edge most aligned with the magnetic force vector was set as the parallel edge and normalized to the total number of observed edges. Connection stability was computed if an edge was detected in at least 1 of the time windows, where the stability value was the total number of occurrences normalized to the total observations.

Magnetic field and force mapping

Nanomagnetic forces are generated through permanent external magnetic field gradients. To induce forces, we used a permanent magnetic field assay designed for generating magnetic fields over standard cell cultures[3]. To provide force estimates, finite element modeling with COMSOL Multiphysics 5.6 was used to predict the force exerted by the MNPs following previous studies[3–5]. Briefly, 8 permanent neodymium magnets (12.7 mm, 476 mT peak) were arranged in an octagonal manner with specific directions for magnetization. The magnetic field interactions were simulated over the assay to obtain the magnetic field (B), and force was approximated through:

$$F = \frac{V_p \chi_p}{\mu_0}(B \cdot \nabla)B eq. 1$$

Where V_p is the volume of magnetically susceptible material in the MNP, χ_p is the magnetic susceptibility of the particle, and μ_0 is the magnetic permeability constant. For simulations, we use a radius of 300 nm to approximate cumulative forces exerted by MNPs and a susceptibility of 1.152 following previous studies[3,6].

Statistical Analysis

All datasets within this study were found to be significantly drawn from normal distributions (Kolmogorov-Smirnov), thus we found the parametric assumption valid for testing. Given the substantial variance in standard deviation across neuronal responses (Figure 3-4), the Welch's correction t-test was used with data reported in text as mean±standard deviation. Data collection and analysis were not performed blind to the experimental conditions. Statistical significance was evaluated at $p \leq 0.05$. Data was not outlier-filtered. Bar plots are reported with bar at mean and error bar as standard deviation if not specified. Box plots are reported with Tukey whiskers and outliers plotted.

Long-term magnetic guidance assay for microelectrode arrays.

To maintain magnetic forces over extended cell culturing, we designed an in-house magnetic ring setup following previous work[3]. To maintain sterilization, the setup was 3d printed with acrylonitrile styrene acrylate (ASA) which could withstand UV and ethanol sterilization for multiple uses. The setup consisted of a baseplate and a magnetic ring to sit around the microelectrode array (Figure S1). The baseplate contained an embedded region to hold the MEA with a hollow center for imaging within the magnetic field. The MEA locks within in this region to allow the magnetic ring to sit flush on the top of the microelectrode array. A gas-permeable lid is used to reduce evaporation in the MEA which is held in place by 3d printed cap. For long-term experiments, the magnetic field is maintained by twist-lock mechanism, using the small arms on the baseplate to hold it together. Orientation was retained by following the cut indent in the baseplate as the bottom of the MEA, which is oriented to the MEA by reference electrode, and finally matched with the triangle on the magnetic ring.

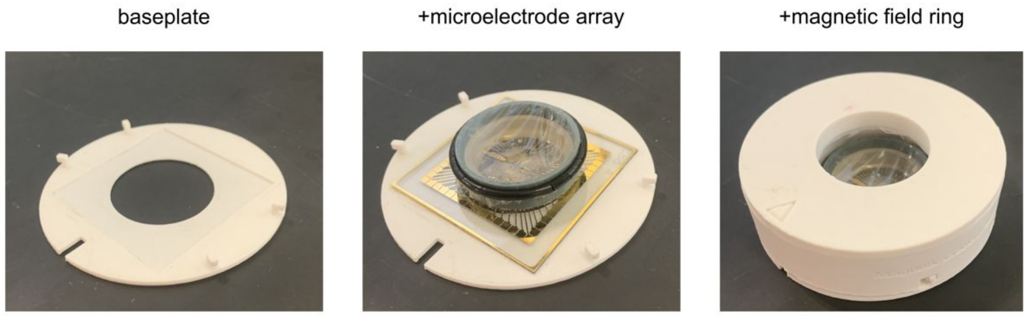

Figure S1. Platform for iNMF guided networks over microelectrode arrays.

Finite element modeling of the magnetic fields and nanomagnetic forces

Nanomagnetic forces are induced through magnetic field pulling of magnetic nanoparticles. The physical principles of the manipulation of magnetic nanoparticles have been previously described in great detail[7–10]. In brief, a magnetically susceptible particle translates electromagnetic energy into kinetic energy through either thermal dissipation or mechanical forces. During magnetic field exposure, magnetic nanoparticles align magnetic domains with the magnetic field. High frequency oscillations of the magnetic field then generate significant thermal dissipation from the particle vibrations induced through domain switching. However, at lower frequencies, particle response to magnetic fields can produce torque on the environment or induce particle translation. This particle translation is then experienced by the surrounding environment as a force. Particle translation through force is driven parallel to the gradient of the magnetic field and scaled by the particle properties and the observed magnetic field potential (eq. 1). This can be visualized as a ball on a hill, where the ball finds the lowest potential energy by rolling down the steepest possible slope.

Propagating magnetic gradient forces over large spaces has considerable challenge given the radial decay of electromagnetic energy. To reduce the force drop-off, magnetically permeable substrates employed across assays warp the local magnetic field, inducing regions of high gradient magnetic fields[5,11]. However, such forces are inconsistent across large-networks and are difficult to implement due to cleanroom biocompatibility issues. Therefore, in this work we aimed to use purely external magnetic fields. Magnetic gradients are an innate property of magnetic sources, dependent on the magnetic field strength, therefore we opted for the strongest reasonable magnets which we could fit within the assay (N52, 476 mT peak, 12.7 mm). To understand the local interactions of magnetic gradient forces, we simulated the magnetic field interactions to approximate the magnetic forces (Figure S2, see Materials and Methods: Magnetic field and force mapping). Adding neighboring magnets manipulated the local magnetic gradient to generate stronger magnetic particle forces that maintained above the femtonewton threshold at further distances, indicating localizing magnetic sources could be used to manipulate the strength of magnetic gradient forces on magnetic nanoparticles.

We then expanded the finite element models by employing magnets circuilarly wrapped to sit outside of the microelectrode array (Figure S3a). Given the electrode region was the primary location of interest, we highlight the two magnetic fields used in Figure 3 of the text. While mag 1 exhibited a stronger magnetic field gradient, mag 2 generated more force through B-field strength (Table S1.

Table S1: Magnetic field interactions to generate forces

Magnetic design	B-field (mT)	∇B (T/m)	Force (pN)
**mag 1**	22.3±4.1	0.16±0.02	0.20±0.05
**mag 2**	83.1±1.8	0.08±0.02	0.40±0.06

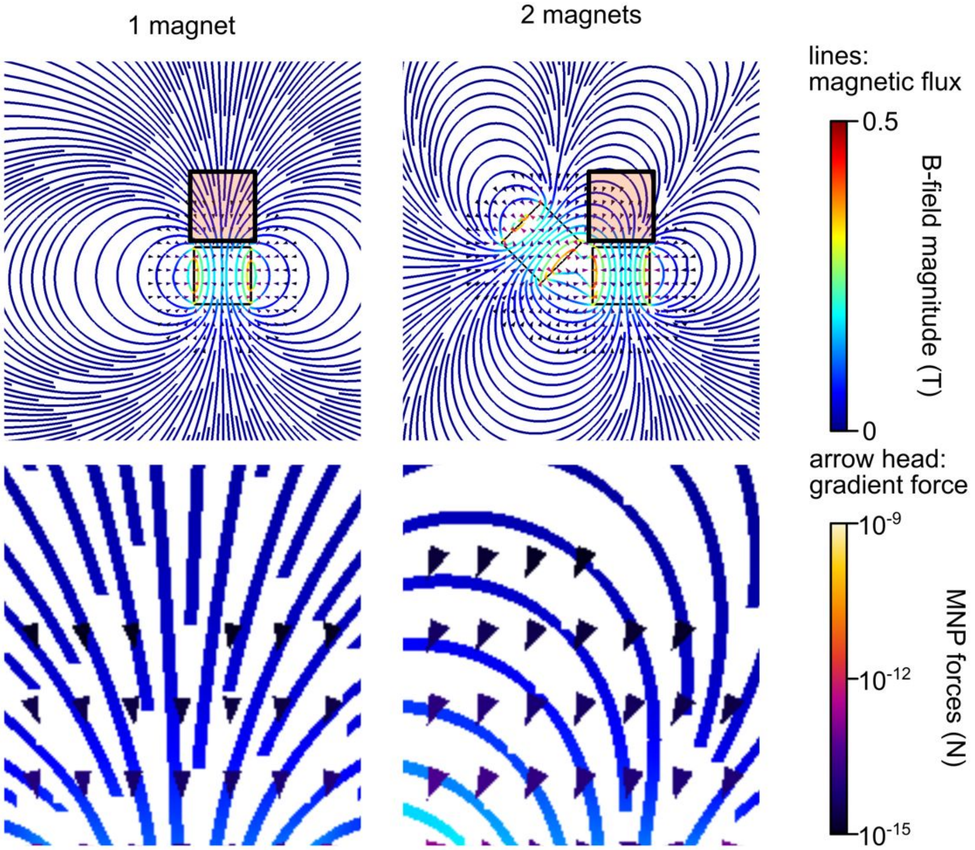

Figure S2. Magnetic source interactions amplify gradient forces.

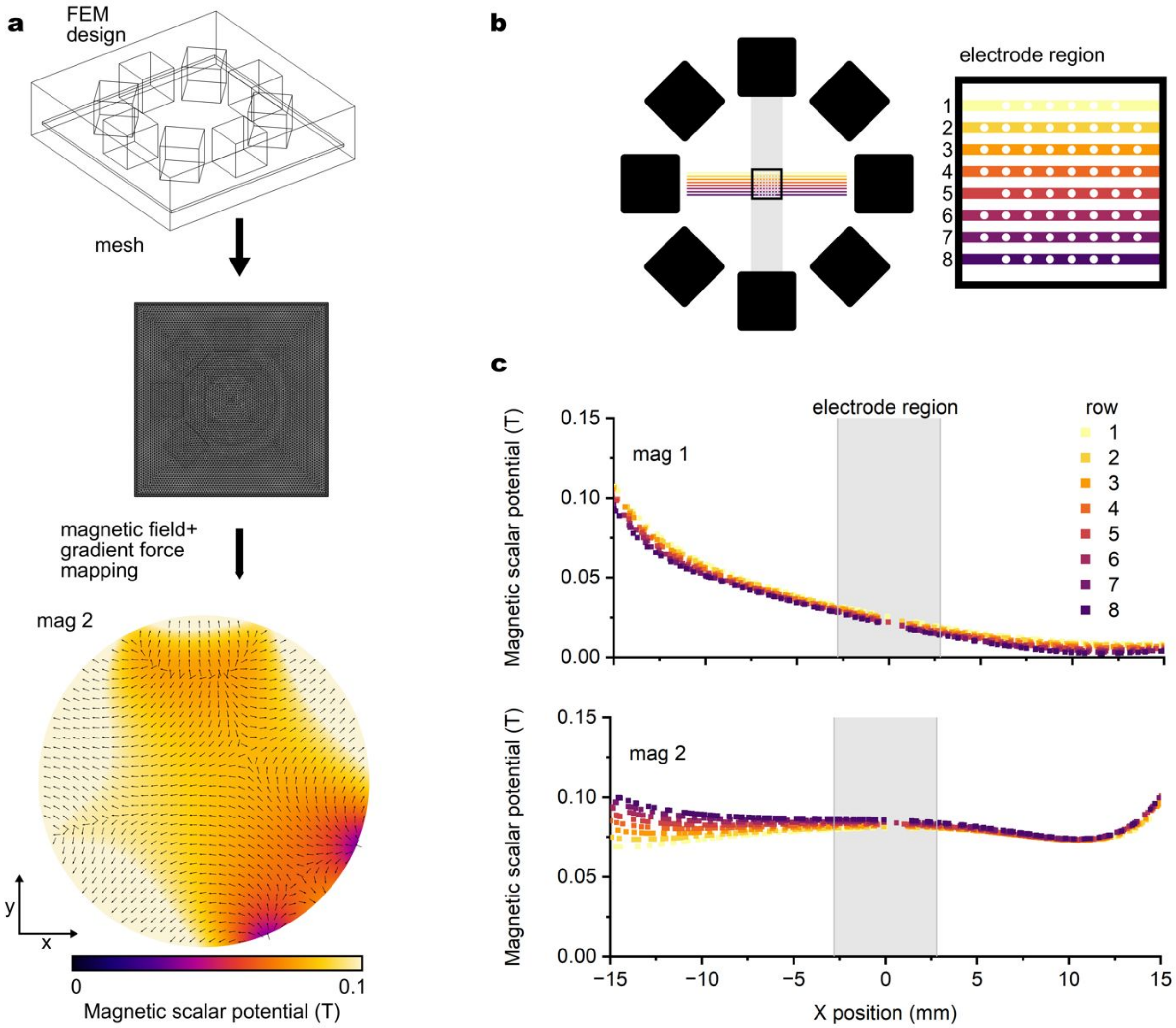

Figure S3. Magnetic field characterization.

(a) Magnetic fields and corresponding forces were predicted through FEM simulations in COMSOL Multiphysics 5.6. (b) To characterize differences in magnetic potential across linear magnetic field designs, line plots across the magnetic region were extracted at the y-center of each microelectrode array. (c) Minimal variance was observed across the electrode region with mag 1 and 2 indicating forces within the electrode region were minimally variable across the y-direction.

afMNP particle characterization.

Neuronal uptake of nanoparticles is controlled by the physical properties of the nanoparticles[12]. To understand the afMNP potential for uptake, we characterized the radius and surface charge, both known to mediate uptake[13]. Nanoparticles were suspended in culture media (10^6 particles/mL), sonicated for 30 min (44 kHz, 40 ºC) and immediately measured with a dynamic light scattering (DLS) analyzer (Mobius, Wyatt Technology) for radius and surface potential. DLS showed particles ranging from 40-100 nm radius which suggested minimal particle clustering following sonication. Zeta potential measurements indicate surface potential remains neutral in culture media.

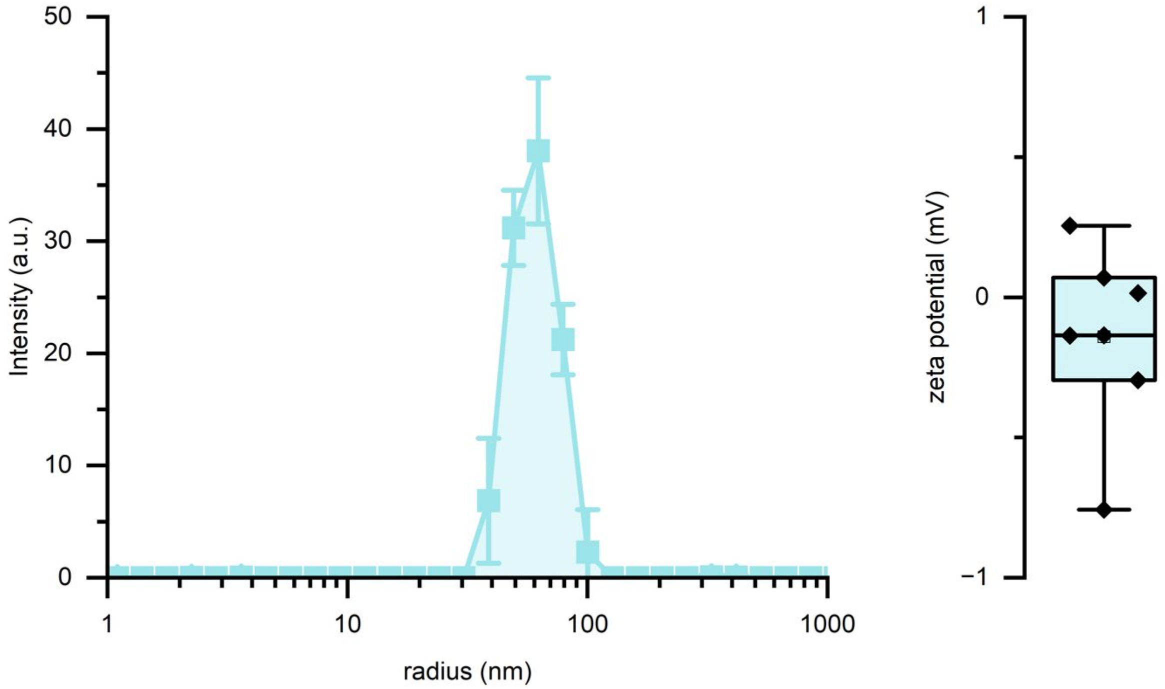

Figure S4. DLS measurements of afMNP radius and zeta potential.
Nanoparticles (10^6 particles/mL) sonicated in culture media match vendor radius specifications and show neutral surface charge (N=7 independent measurements).

1 DIV cortical neurons internalize afMNPs by 2 h exposure

Axonal specification through forces has been correlated to the remote pulling of cell-internalized magnetic nanoparticles[5]. Therefore, we sought to measure afMNP uptake in cultured neurons. Cortical neurons were plated on PDL coated glass bottom dishes (1000 cells/mm^2) and cultured for 24 h. Sonicated (44 kHz, 40 ºC) afMNPs in culture media were added to the neurons (10^{12} particles/ml) and incubated for 2 h. Using high-resolution oil immersion (1500x, NA=1.52) microscopy with z-stacks (0.18 µm step), afMNP colocalization was performed across neuron cultures (n=3 cultures). The afMNps were observed both membrane-bound and internalized at the 2 h mark, indicating forces could be evoked within the cytosol. Microglia were also observed to have substantial interactions with the afMNPs.

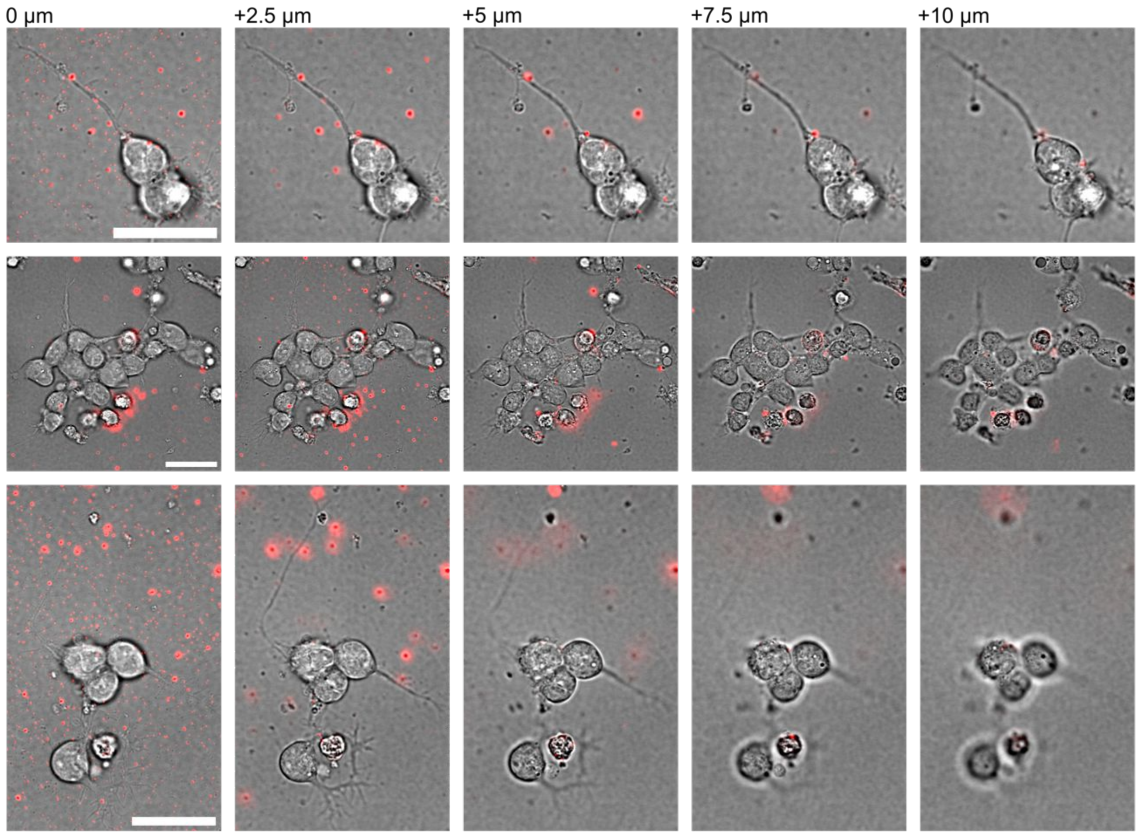

Figure S5. Supporting 2h afMNPs z-stacks.
Representative false color merged z-stack images (Gray: DIC; Texas Red: afMNPs) of cortical neurons at 1 DIV following 2h exposure to afMNPs show both afMNP interactions and neuronal uptake (scalebar: 20 µm). Each row is a representative image from an independent culture.

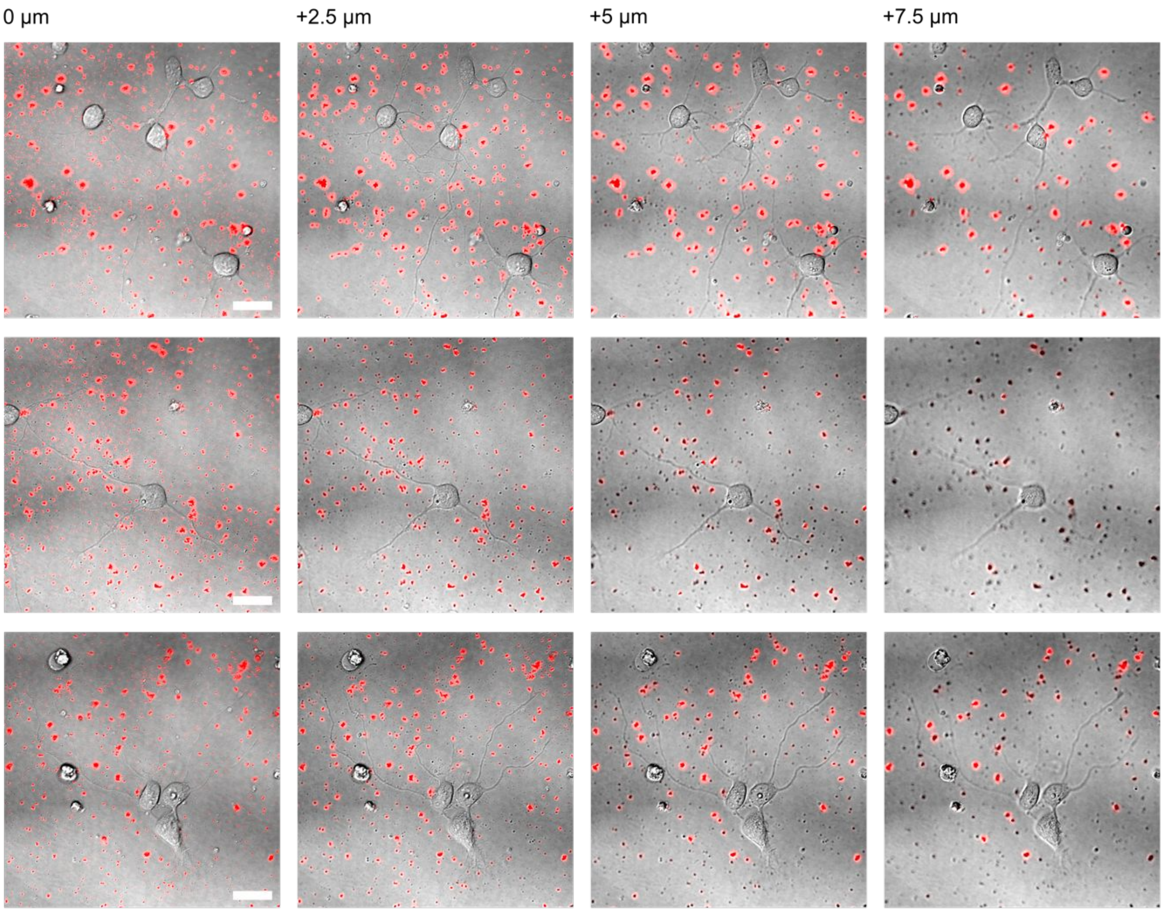

Figure S6. Supporting 24h afMNPs images.
Representative false color merged z-stack images (Gray: DIC; Texas Red: afMNPs) of cortical neurons at 2 DIV following 24h exposure to afMNPs show both afMNP interactions and neuronal uptake (scalebar: 20 µm). Each row is a representative image from an independent culture.

210

Enhanced neurite outgrowth is dependent on force guidance

To ensure the nanomaterials alone did not impact neurite outgrowth, we contrasted neurite length of control cultures (no MNP + no Mag), cultures exposed to afMNPs but no force (MNP + no Mag), and neurites exposed to either magnetic field 1 or 2 (MNP + mag 1 or 2). To contrast neurite length, we used Kruskal-Wallis ANOVA to identify differences (p<0.0001 was found at each timepoint) with post-hoc Dunn's test at each time point and reported below.

At 2 DIV, control neurites exhibiting a median length of 12.1 µm, while neurites under afMNP only exposure significantly (p=0.0234) increased median length to 17.0 µm and nanomagnetic guidance exhibited significant increases in median length with mag 1 and 2 enhancing median neurite length to 22.0 µm (p<0.0001 for control and p=0.0005 for afMNP, no force) and 21.4 µm respectively (p<0.0001 for control and p=0.0035 for afMNP, no force), with no significant difference between the two (p=1.0000). At 5 DIV, no significance was found between control and afMNP, no force cultures (p=0.0679), and a significant increase in both nanomagnetic force guidance parameters (p<0.0001 for both). By 8 DIV, we observed significantly longer neurites in afMNP, no force cultures of 32.6 µm median neurite length while control cultures exhibited a median neurite length of 23.6 µm (p=0.0004). Both were found to be significantly less than the median neurite length of nanomagnetic guided cultures with mag 1 exhibiting 39.8 µm (p<0.0001 for control and p=0.0106 for afMNP, no force) and mag 2 exhibiting 39.5 µm lengths (p<0.0001 for control and p=0.0019 for afMNP, no force) with no significant difference between the two (p=1.0000). Finally, by 14 DIV, control cultures exhibited a median of 35.4 µm while afMNP, no force cultures were not significantly different with a median 32.9 µm neurite length. Here, nanomagnetic forces in mag 1 exhibited a median neurite length of 41.6 that was significantly greater than the afMNP, no force cultures (p=0.0134) but not significantly greater than control cultures (p=0.8162). Nanomagentic forces under mag 2 showed significant increases in neurite length to a median of 44.2 µm which was significantly greater than control (p=0.0033) and afMNP, no force (p=0.0001) but not mag 1 (p=0.1399).

In summary, afMNP exposure without magnetic forces minimally mediates neurite outgrowth, thus suggesting nanomagnetic force guidance is responsible for longer neurites.

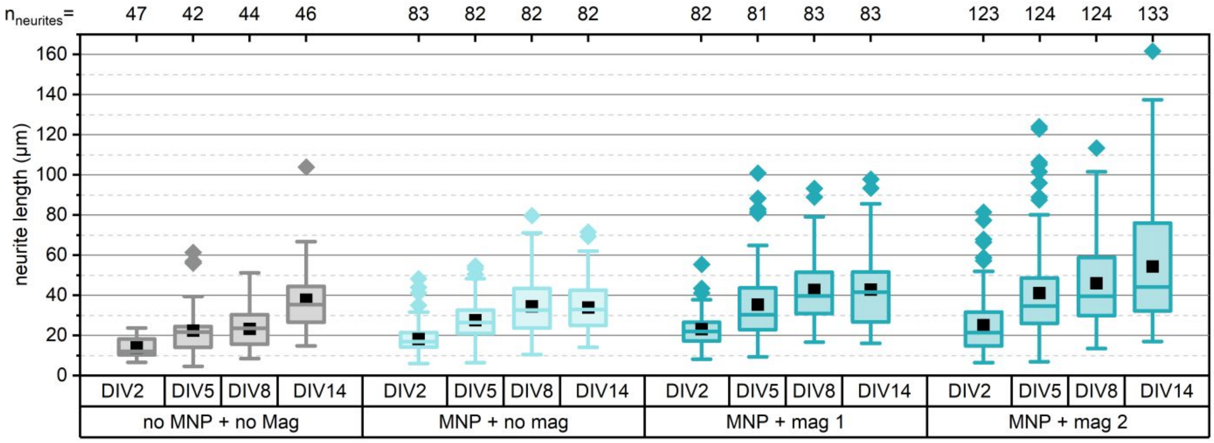

Figure S7. Force mediation of afMNPs required for enhanced neurite growth.
Mean neurite length measured from cortical neurons grown on the microelectrode arrays over the force mediation exhibited longer neurites than control samples under no afMNP exposure or with afMNP exposure.

Neurite directionality detection through hough transform

To mitigate bias from neurite selection during tracing, we used automatic image processing methods to further confirm directionality. Following 14 DIV characterization of networks, we used a fluorescent calcium stain (Fluo 4-AM, 1:1 v/v, 1 h incubation) to produce contrast of neurons on the MEA for image processing methods. Fluorescent images were acquired at 200x (Leica DMI-8) and processed in post. In brief, fluorescent images were imported into MATLAB 2022A through the imread function. The image was normalized to a max intensity of 1 and a binary threshold of 0.007 was set to remove background noise. The internal hough function was then called on the binarized image. Consecutively, the internal houghpeaks and houghlines functions were called to locate key linear elements in the image. The theta value where a peak occurs represents the orientation of the line relative to the x-axis. Therefore, we used these theta values as the orientation of a neurite within the image to establish alignment.

To characterize the method, we traced neurites within an image through Simple Neurite Tracer (Figure S6a) and found the directional vectors from the start and end point (Figure S6b). Then, we processed the image with the hough transform, finding a significant frequency of points at the 45° orientation (Figure S6c). As the hough transform is unable to determine directionality of lines, only orientation, this result corresponds well to the high frequency of neurites observed in Q1/Q3 in Figure S6b. We then expanded this across force regions within the network (Figure S6d-f). Regions 1 and 4 followed such alignment, which were localized in high force (>4 pN) regions.

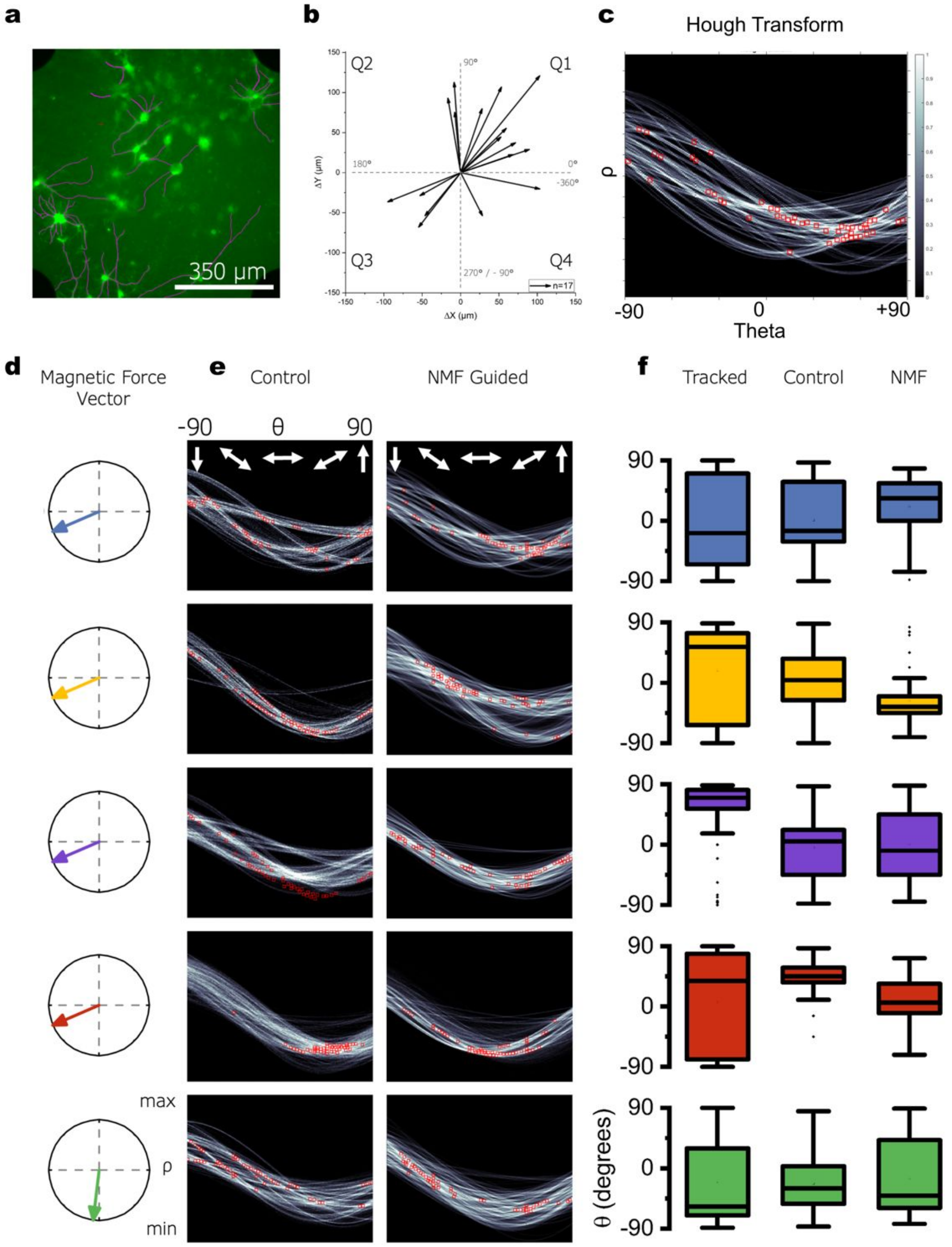

Figure S8. Hough transform reveals neurite alignment with the magnetic field.
(a) False color fluorescent image of 14 DIV cortical cells stained with Fluo-4AM enhanced neurite tracing. (b) SNT neurite tracing output vectors highlights neurites in mostly aligned with quadrant 1 and 3. (c) Hough transform performed on the same images locates lines within images by cross-over detection (small red squares). Neurite directionality is lost in the Hough transform, suggesting neurites aligned in quadrants 1 and 3 are both contained in the 0º to 90º range, centered at 45º while perpendicular lines in quadrants 2 and 4 occur in -90º to 0º range. (d) Simulated magnetic force vector. (e) Hough Transforms were performed on contrast enhanced fluorescent images showing the neuronal network (E18 cortical neurons, 14 DIV, calcium stained) in the five different ROIs. (f) Box plots show angle distribution of directional neurite growth.

Cytotoxicity assay

Neuron-nanoparticle interactions, especially with magnetic materials and cell uptake, can cause cytotoxicity issues. Further, applying mechanical forces to neurons could cause cell damage through membrane interactions. To ensure the viability of networks within our experimental protocol, we performed a Live/Dead assay (Invitrogen, R37601) on cortical neuron networks across experimental variables on 14 DIV. The BOBO-3 Iodide dead stain showed no evidence of membrane rupture under nanomagnetic forces (Figure S9a). There was no significant evidence of changes in cell viability across parameters (n=3 samples per network, n=3 independent cultures per parameter; 2-way ANOVA, magnetic field: p=0.4004; afMNPs: p=0.21903).

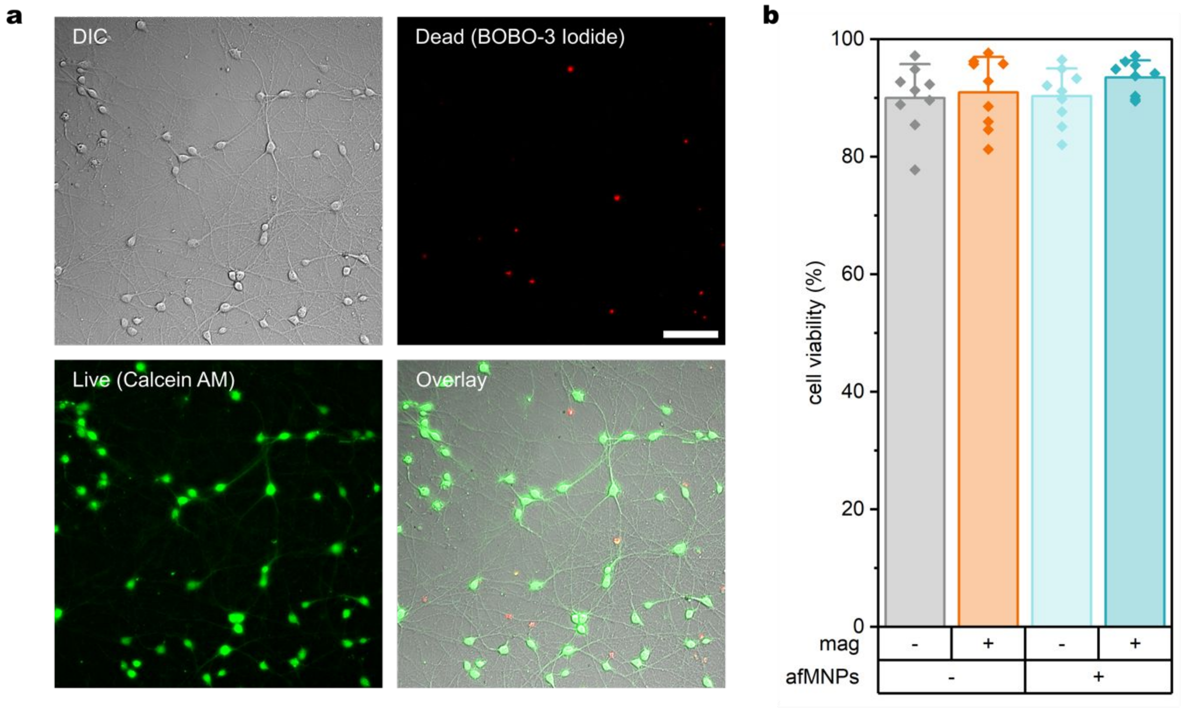

Figure S9. Cytotoxicity assay

(a) False color fluorescent Live/Dead image of 14 DIV nanomagnetic force exposed cortical cells stained with Calcien AM and BOBO-3 Iodide. (b) Viability (% of live cells over total cells) of 14 DIV cultures exposed to experimental parameters. (n=3 samples per network, 3 independent networks per parameter).

Activity profiling in developing networks

Cortical networks organize into functional networks during development *in vitro*. We tracked spontaneous neuronal activity (4 min) in control and nanomagnetic guided networks from 8-14 DIV to provide insight into the functional attributes of the networks. Microelectrode signals were processed with bandpass filtering (300-4000 Hz) and falling edge spike detection (5 std) to extract spikes. Electrodes without spikes were omitted from further analysis. Spiking activity from the same network exhibited various states across the recording windows (Figure S10a-b). We quantified features of spiking activity through firing rate, interspike interval, burst rate, and the number spikes per burst (Figure S10c-f). Mean firing rate, interspike interval, and mean burst rate showed significant relationships to DIV but not guidance parameters, while the mean spikes per burst showed a significant interaction between guidance parameters but not over DIV (Table S2).

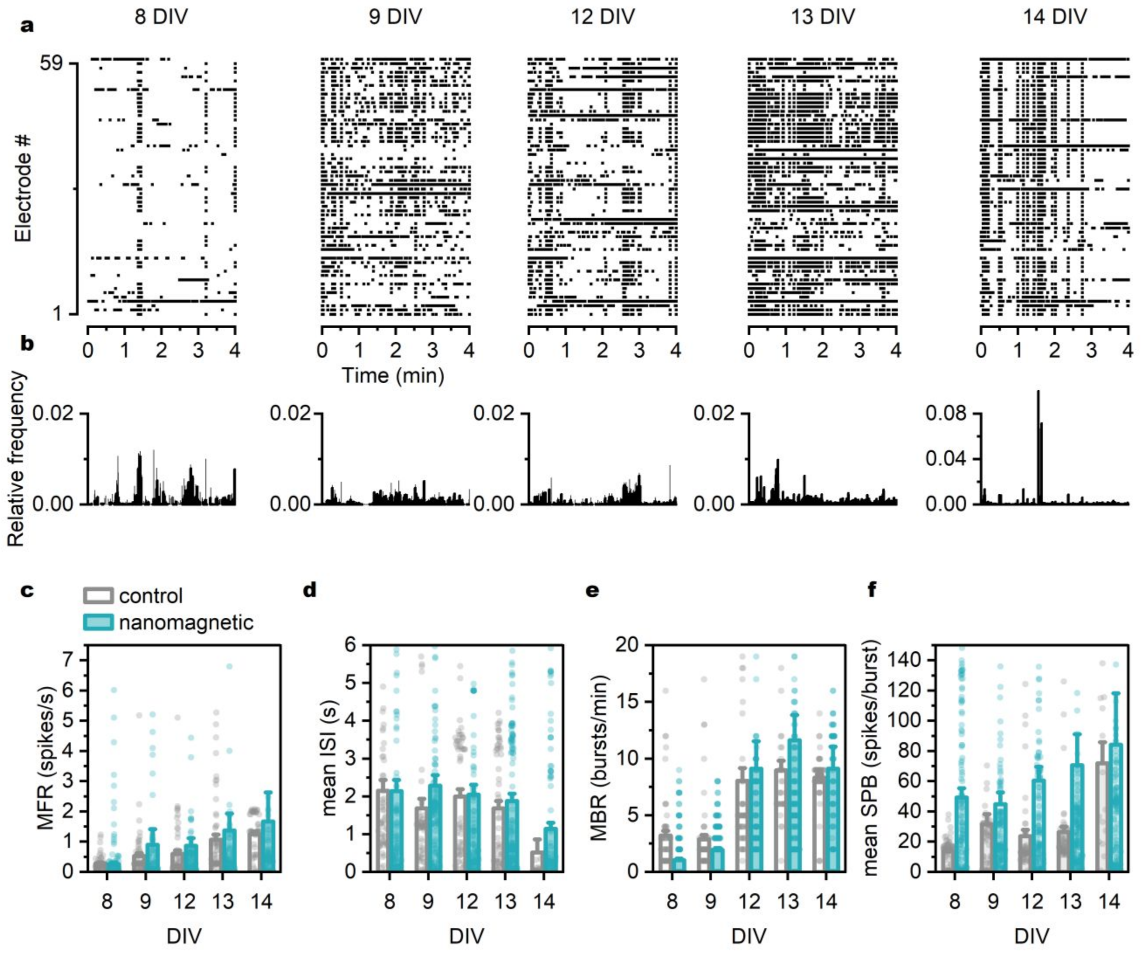

Figure S10. Neuronal activity progression during growth

(a) Spike raster plots of example nanomagnetic guided network (+afMNPs, +magnetic field) grown on the microelectrode arrays with recordings over network formation. (b) Relative frequency of spikes at 1s intervals. (c-f) Functional parameters measured over the network formation phase of control (- afMNPs, -magnetic field) and nanomagnetic (+afMNPs, +magnetic field) guided networks (177 electrodes over 3 independent cultures for each parameter). (c) Mean firing rate (MFR) (d) Mean interspike interval (e) Mean burst rate (f) Mean spikes per burst.

Table S2: 2-way ANOVA *p*-values for comparing the functional features of guided and control networks

	**MFR**	**ISI**	**MBR**	**SPB**
**Control vs. nanomagnetic**	<0.0001	0.12421	0.92536	0.04371
**`DIV**	0.0001	<0.0001	<0.0001	0.40262

Graphical representation of nearest neighbor measurements for granger causality metrics

To characterize the functional connectivity of cortical circuits, we used a pre-packaged granger causality tool (GCCA[2]; see Materials and Methods: Granger causality-based electrophysiological signal processing for more information). As the cultured neuronal networks were planar, functional information must be propagated linearly across the network. Therefore, we chose to only measure nearest neighbors for each granger causality metric. This approach allowed us to measure causality within a central electrode, and up to 8 corresponding neighbors to determine functionality. Causal inference was drawn from 1 min windows to establish network topology and the functional dynamics over time.

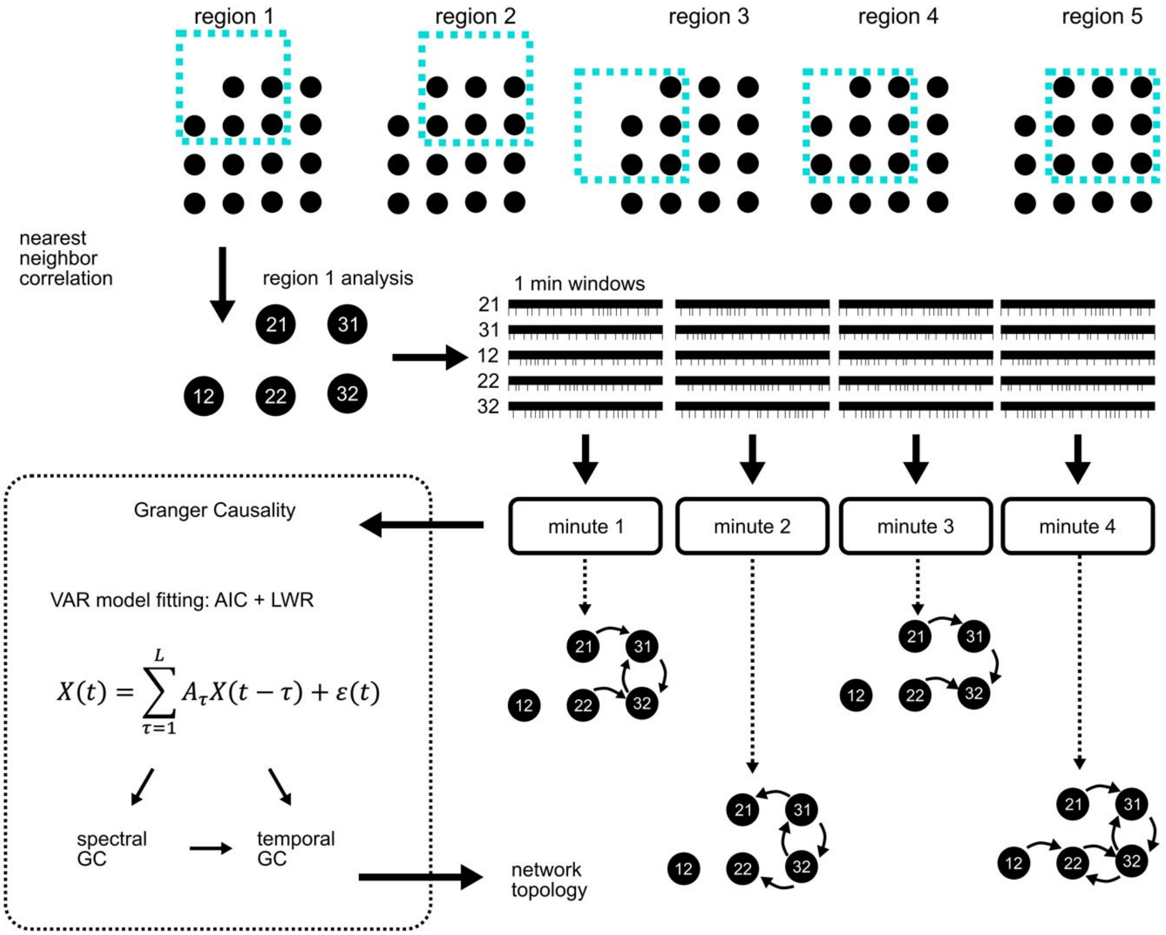

Figure S11. Granger causality signal processing.
(a) Nearest-neighbor approach to signal correlation. To achieve this, a small window of electrodes (3x3 grid) was used to correlate the network at a given time. The 4 min recording from each electrode was decomposed into 1 min windows for GC measurement. The raw electrode signal was used to reduce inaccuracies[2]. GC was computed by contrasting against vector autoregressions (VAR) fit to the dataset. GCCA implements a spectral causality which is integrated into the temporal GC to define significant directional correlation. If electrodes share a causal connection ($p<0.05$), an edge is mapped with an arrow.

Supplemental video descriptions

Supplemental Video 1-2: Timelapse differential interference contrast (400x total magnification) videos of independent cultures of cortical neurons before (0-120 min) and during (121-301) nanomagnetic force guidance. Neurons were cultured on glass bottom petri dishes pre-coated with PDL (0.05 µg/mL) to 1 DIV and exposed to afMNPs (10^{12} particles/mL) for 24 h before imaging. Neurons were maintained in a stage-top chamber with a manual gas mixer during imaging (Okolab, 37 ºC, 5% CO_2).

References

(1) Arshadi, C.; Günther, U.; Eddison, M.; Harrington, KyleI. S.; Ferreira, TiagoA. SNT: A Unifying Toolbox for Quantification of Neuronal Anatomy. *Nat Methods* **2021**, *18* (4), 374–377. https://doi.org/10.1038/s41592-021-01105-7.

(2) Seth, A. K. A MATLAB Toolbox for Granger Causal Connectivity Analysis. *J Neurosci Methods* **2010**, *186* (2). https://doi.org/10.1016/j.jneumeth.2009.11.020.

(3) Judge, D.; Kunze, A. Neural Network Growth under Heterogenous Magnetic Gradient Patterns. In *2019 9th International IEEE/EMBS Conference on Neural Engineering (NER)*; IEEE, 2019; pp 191–194. https://doi.org/10.1109/NER.2019.8716902.

(4) Tay, A.; Kunze, A.; Murray, C.; Di Carlo, D. Induction of Calcium Influx in Cortical Neural Networks by Nanomagnetic Forces. *ACS Nano* **2016**, *10* (2), 2331–2341. https://doi.org/10.1021/acsnano.5b07118.

(5) Kunze, A.; Tseng, P.; Godzich, C.; Murray, C.; Caputo, A.; Schweizer, F. E.; Di Carlo, D. Engineering Cortical Neuron Polarity with Nanomagnets on a Chip. *ACS Nano* **2015**, *9* (4), 3664–3676. https://doi.org/10.1021/nn505330w.

(6) Kunze, A.; Tseng, P.; Godzich, C.; Murray, C.; Caputo, A.; Schweizer, F. E.; Carlo, D. Di. Engineering Cortical Neuron Polarity with Nanomagnets on a Chip. **2015**. https://doi.org/10.1021/nn505330w.

(7) Suwa, M.; Uotani, A.; Tsukahara, S. Magnetic and Viscous Modes for Physical Rotation of Magnetic Nanoparticles in Liquid under Oscillating Magnetic Field. *Appl Phys Lett* **2020**, *116* (26). https://doi.org/10.1063/5.0010095.

(8) Surpi, A.; Shelyakova, T.; Murgia, M.; Rivas, J.; Piñeiro, Y.; Greco, P.; Fini, M.; Dediu, V. A. Versatile Magnetic Configuration for the Control and Manipulation of Superparamagnetic Nanoparticles. *Sci Rep* **2023**, *13* (1). https://doi.org/10.1038/s41598-023-32299-9.

(9) Wei, W.; Wang, Z. Investigation of Magnetic Nanoparticle Motion under a Gradient Magnetic Field by an Electromagnet. *J Nanomater* **2018**, *2018*. https://doi.org/10.1155/2018/6246917.

(10) Shevkoplyas, S. S.; Siegel, A. C.; Westervelt, R. M.; Prentiss, M. G.; Whitesides, G. M. The Force Acting on a Superparamagnetic Bead Due to an Applied Magnetic Field. *Lab Chip* **2007**, *7* (10), 1294–1302. https://doi.org/10.1039/B705045C.

(11) Tseng, P.; Judy, J. W.; Di Carlo, D. Magnetic Nanoparticle-Mediated Massively Parallel Mechanical Modulation of Single-Cell Behavior. *Nat Methods* **2012**, *9* (11). https://doi.org/10.1038/nmeth.2210.

(12) Ma, N.; Ma, C.; Li, C.; Wang, T.; Tang, Y.; Wang, H.; Mou, X.; Chen, Z.; He, N. Influence of Nanoparticle Shape, Size, and Surface Functionalization on Cellular Uptake. *Journal of Nanoscience and Nanotechnology*. 2013. https://doi.org/10.1166/jnn.2013.7525.

(13) Behzadi, S.; Serpooshan, V.; Tao, W.; Hamaly, M. A.; Alkawareek, M. Y.; Dreaden, E. C.; Brown, D.; Alkilany, A. M.; Farokhzad, O. C.; Mahmoudi, M. Cellular Uptake of Nanoparticles: Journey inside the Cell. *Chem Soc Rev* **2017**, *46* (14), 4218–4244. https://doi.org/10.1039/C6CS00636A.
